# Effective behaviour change techniques for physical activity and healthy eating in overweight and obese adults; systematic review and meta-regression analyses

**DOI:** 10.1186/s12966-017-0494-y

**Published:** 2017-03-28

**Authors:** Gro Beate Samdal, Geir Egil Eide, Tom Barth, Geoffrey Williams, Eivind Meland

**Affiliations:** 1 Department of Global Public Health and Primary Care, University of Bergen, Kalfarveien 31, N-5018 Bergen, Norway; 2 Department for Research and Development, Haukeland University Hospital, Bergen, Norway; 3 Centre for Clinical Research, Haukeland University Hospital, Bergen, Norway; 4 Member of Motivational Interviewing Network of Trainers (MINT), Allasso, Norway; 5 School of Medicine, University of Rochester, Rochester, NY USA

**Keywords:** Systematic review, Behaviour change techniques, Healthy eating, Physical activity, Meta-regression, Heterogeneity, Self-regulation

## Abstract

**Purpose:**

This systematic review aims to explain the heterogeneity in results of interventions to promote physical activity and healthy eating for overweight and obese adults, by exploring the differential effects of behaviour change techniques (BCTs) and other intervention characteristics.

**Methods:**

The inclusion criteria specified RCTs with ≥ 12 weeks’ duration, from January 2007 to October 2014, for adults (mean age ≥ 40 years, mean BMI ≥ 30). Primary outcomes were measures of healthy diet or physical activity. Two reviewers rated study quality, coded the BCTs, and collected outcome results at short (≤6 months) and long term (≥12 months). Meta-analyses and meta-regressions were used to estimate effect sizes (ES), heterogeneity indices (I^2^) and regression coefficients.

**Results:**

We included 48 studies containing a total of 82 outcome reports. The 32 long term reports had an overall ES = 0.24 with 95% confidence interval (CI): 0.15 to 0.33 and I^2^ = 59.4%. The 50 short term reports had an ES = 0.37 with 95% CI: 0.26 to 0.48, and I^2^ = 71.3%. The number of BCTs unique to the intervention group, and the BCTs goal setting and self-monitoring of behaviour predicted the effect at short and long term. The total number of BCTs in both intervention arms and using the BCTs goal setting of outcome, feedback on outcome of behaviour, implementing graded tasks, and adding objects to the environment, e.g. using a step counter, significantly predicted the effect at long term. Setting a goal for change; and the presence of reporting bias independently explained 58.8% of inter-study variation at short term. Autonomy supportive and person-centred methods as in Motivational Interviewing, the BCTs goal setting of behaviour, and receiving feedback on the outcome of behaviour, explained all of the between study variations in effects at long term.

**Conclusion:**

There are similarities, but also differences in effective BCTs promoting change in healthy eating and physical activity and BCTs supporting maintenance of change. The results support the use of goal setting and self-monitoring of behaviour when counselling overweight and obese adults. Several other BCTs as well as the use of a person-centred and autonomy supportive counselling approach seem important in order to maintain behaviour over time.

**Trial Registration:**

PROSPERO CRD42015020624

**Electronic supplementary material:**

The online version of this article (doi:10.1186/s12966-017-0494-y) contains supplementary material, which is available to authorized users.

## Background

Health behaviour, such as physical inactivity, unhealthy eating, smoking and excessive alcohol consumption, are leading contributors to morbidity and premature mortality in Europe, due to the development of non-communicable diseases (NCDs). The World Health Organization (WHO)’s Global Action Plan urges national governments to develop NCD targets and public health strategies to improve people’s health [1]. Obesity is associated with several risk factors, and many studies target weight loss as a primary outcome although it is difficult to maintain weight loss over time. Moreover, weight neutral interventions that encourage body acceptance, combined with healthy behaviour and wellbeing, can improve health without targeting weight loss [2].

There is a growing interest in the use of theories of behaviour change and a total of 83 theories are identified [3]. Theories like social cognitive theory, theory of planned behaviour, and the transtheoretical model explain why people adopt a behaviour, but provides little explanation of how the initiation and maintenance of behaviour might differ. A person’s self-regulatory strength is a limited, but renewable cognitive resource. Over time, people who are motivated by their own needs and desires, find it easier to sustain the new behaviour [4]. Thus, the determinants of behaviour may differ across the different phases of the behaviour change process. Consequently, intervention techniques that help people initiate change may not necessarily have the same effect on behaviour maintenance. In accordance with this, a review summarizing 100 theories that explain maintenance of behaviour change, have identified five overarching theoretical themes, among them positive maintenance motives, and active self-regulation [5].

Behaviour change interventions use different strategies and behaviour change techniques (BCTs) to support a participant’s self-regulation skills and resources in the change process. A BCT is defined as the smallest “active ingredient” of an intervention [6]. Recent developments within science of behaviour change has led to the definition of the first 26 BCTs, later 44 BCTs, and recently 93 internationally agreed and validated BCTs (the Behaviour Change Technique Taxonomy version1, BCTTv1) [6–8]. Several reviews have tested the associations between BCTs and the intervention effect. Michie and colleagues’ study revealed no significant associations between BCTs and study effects concerning physical activity (PA) and improved diet [9]. The BCT self-monitoring of behaviour explained the greatest between-study heterogeneity. Nor did Dombrowski and colleagues, find significant associations between BCTs and PA outcomes [10], but the BCT providing instruction on how to perform the behaviour was associated with improved diet outcomes. McDermott and colleagues found no positive association whatsoever, but the BCT providing feedback had a significant negative effect [11]. Williams and colleagues reported that the BCTs action planning, providing instructions, and reinforcing efforts towards behaviour were associated with higher levels of PA [12]. Lastly, Lara and colleagues found the BCTs barrier identification and problem solving, planning of social support, and setting goals for outcome results, providing feedback, and the use of prompts, e.g. put a sticker on the refrigerator, supported better diet outcome results [13].

The evidence that theory based interventions leads to better outcomes is inconsistent [14–16]. However, using a number of BCTs congruent with Control Theory, have been associated with increased intervention effects, e.g. through combining self-monitoring of behaviour with goal setting, providing feedback on performance, and review of behaviour goals [9, 10].

Behaviour change interventions may also have different therapeutic approaches, e.g. Cognitive behaviour therapy (CBT), or Acceptance and commitment therapy (ACT) or be delivered by professionals using a certain communication style. Motivational interviewing (MI) is a client-centred method for enhancing intrinsic and autonomous motivation to change, and is often used synonymously with person-centered counselling. The taxonomies define the counselling methods as a separate BCT. In some studies MI based counselling has not been associated with intervention effects [10, 13], and Dombrowski and colleagues concluded that volitional planning and action strategies are more effective than MI [10]. Therefore, successful behaviour change may dependent more on volitional and positive motivation and self-regulation skills.

Self-determination theory (SDT) is one of the many theories that explain maintenance of change [5]. SDT claims that successful increases in physical activity or healthy eating are not maintained over time if the reasons for doing so are mostly controlling, e.g. external pressure. Evidence based on SDT suggests that health personnel may enhance their efficacy by positively influencing clients’ motivation and thus, make the behaviour become more autonomously regulated and valued [17–19]. Conceptual overlap and similarities exist between the techniques in MI and interventions based on SDT. SDT based interventions often use MI techniques in counselling and SDT can help explain why MI works [20, 21].

Building on these theoretical assumptions, there is a need to provide further insight on the utility of BCTs in facilitating long term behaviour change. Is there a difference in effective BCTs associated with the initiation and maintenance of change? We hypothesized that autonomy supportive counselling emphasizing both self-regulatory BCTs and internal motivation give persistence of change over time. To our knowledge, this is the first systematic review with meta-regression analyses using BCTTv1 to identify effective BCTs for PA and healthy eating among overweight and obese adults, differentiating short and long term follow-up. Our objectives were accordingly to:

1. Synthesize the evidence of behavioural interventions, aiming to improve PA and healthy eating among overweight and obese adults in short and long term, and
2. Examine to what extent intervention effectiveness varies across studies depending on BCTs and other study characteristics.

## Methods

The reporting of this systematic review were performed in accordance with the Preferred Reporting Items for Systematic Review and meta-analysis guidelines (PRISMA) and Template for intervention description and replication (TIDieR) checklist and guide [22, 23].

### Eligibility criteria

Eligible study designs included published, peer-reviewed, randomized and cluster randomized controlled trials (RCTs) of behaviour change interventions providing baseline and/or follow-up data at minimum 12 weeks after randomization. The intervention duration was set at ≥ 12 weeks to allow time for counselling to effect the behaviour change process. The interventions had to promote change in diet and/or PA, compared to usual care, waiting list control or less intensive interventions. Only interventions applying behaviour- and/or cognitive behavioural strategies were included, whereas we excluded simply educational studies, e.g. “giving information”. A mean/median age ≥ 40 years and a BMI ≥ 30 kg/m^2^ were required to recruit participants at risk of developing non-communicable diseases. For pragmatic reasons only papers in Scandinavian or English languages were included. In fact, only English-language articles were identified. There was no restrictions on the types of intervention setting. Main outcomes were objective or subjective behavioural measures of PA and/or diet at baseline, at short term follow-up (≤6 months) and at long term follow-up (≥12 months) when available.

### Search method

The electronic databases MEDLINE, PsycInfo and EMBASE were searched in cooperation with the library service at the Medicine and Dentistry Library, University of Bergen, Norway. Articles published in peer-reviewed journals from January 2007 to April 2013 using a search strategy based on previous systematic reviews [10] with these adjustments were targeted; “Motiv* interview*” was added to the concept “psychological interventions”, the search term “healthy eating” was added to “diet”, and ”physical activity” or “walking” were added to the term “exercise”. Detailed search strategies can be obtained from the author. The initial search was updated once to October 2014. The reference list of relevant reviews on the topic of interest was also screened [19, 24–33]. Additionally, we manually searched the following journals: International Journal of Obesity; International Journal of Behavioural Nutrition and Physical Activity; Obesity Research and Clinical Practice; and International Journal of Behavioural Medicine. We enlisted all references in EndNote X7. The review was preregistered at PROSPERO with protocol and search strategy (CRD 42015020624).

### Data extraction

After removing duplicate publications, we carried out a relevance check of 6283 articles. The first 100 titles were screened in cooperation using a data collection form, and discussed by two reviewers (GBS and EM). In the next step, 100 titles were screened independently two separate times. This procedure yielded 94 and 90% agreement between the reviewers. Disagreements were solved through discussion. Thereafter, identifications of titles were performed by one researcher (GBS). The screening yielded 584 relevant titles of which abstracts were obtained. The first 20 abstracts were screened independently by two reviewers (GBS and EM). Thereafter GBS and EM independently screened all obtained abstracts. There was a 85% agreement whether to 1) include, 2) exclude or 3) carry out a full text evaluation. If the study was an analysis of mediators or a subgroup analysis, we included the main intervention study. We obtained published protocols and published online supplementary materials if available. We also used this approach in data extraction.

Study and intervention characteristics were collected by GBS using two data collection forms and later checked by EM. The data extracted were in accordance with the eight first items of TIDierR checklist for describing an intervention; brief name of the intervention, intervention theory, description of the intervention, procedures (methods), who provided, how, where, when and how much [23]. We were unable to identify the outcome results in nine studies. The authors of six of these papers answered our request for more data; four of them returned the information and two were unable to produce the data. The latter studies were subsequently excluded. If the study targeted both PA and diet, the outcome results were extracted for each behaviour separately.

### Coding behaviour change techniques

When the interventions mentioned “education”, we coded BCT 4.1 instruction on performing the behaviour and 5.1 information on health consequences. When “training” was mentioned, it was coded as BCT 4.1. This approach is previously used by Presseau et al. to acknowledge a minimum of educational strategies in the interventions [34]. A BCT was only coded when there was clear evidence of inclusion, e.g. the BCT had to be directly applied to the target behaviour(s): PA or diet. The 93 BCTs had to be rated as either present (1) or absent (0). Only BCTs identified by both researchers were coded as present. The BCTs in the intervention- and control groups were identified separately, and the BCTs exclusively applied in the intervention group were extracted. Only BCTs present in the intervention and absent in the control condition were thus recorded. This approach was used to explain the difference in effect as emphasized by Peters and colleagues [35], and used by MacDonald and colleagues [36]. In addition, we recorded the total number of BCTs of both intervention arms.

### Coding of other study characteristics

The following characteristics that might influence the intervention effect were extracted: the number of different BCTs in the intervention groups as compared with the control groups; total sum of BCTs in intervention plus control group; duration of intervention in weeks; treatment setting; format of delivery (coded as individual versus group or mixed); source of delivery (coded as community or workplace versus primary care or hospital); theory-based interventions (theory mentioned or not); method-based interventions (coded as MI or SDT versus ACT, CBT, Health-at-every-size (HAES) or Mindfulness based interventions or other method, versus no method mentioned/unclear); and type of outcome data (objective versus self-reported).

### Risk of bias in individual studies

GBS and EM independently assessed risk of under- or overestimating the intervention effects using a standard risk of bias form covering: random sequence generation; allocation concealment; performance bias; blinding of assessment; attrition; and reporting bias [37]. We made judgements according to three categories; “low risk”, “high risk” or “unclear risk”, and disagreements were resolved through discussions.. We evaluated the risk of bias due to the lack of blinding of outcome assessment as «low» when outcomes were objective measures, as for instance in the use of an accelerometer. All diet measurements were self-reported with a high risk of performance bias (except vitamin C in blood in one study).

### Extraction of effects

Where studies employed more than one intervention arm, the most active intervention and the most passive comparison were selected. We collected outcomes at the following time-points if available: 1) at baseline; 2) post intervention (≤ six months after baseline) in order to identify initial change in behaviour; and 3) at last follow up (≥12 months after baseline) in order to identify maintenance of change. (See arguments for these two time points below.) Where the studies reported more than one outcome per behavioural domain, we sought and extracted outcomes in the following order of priority: 1) measures defined as the primary outcomes; 2) objective measurements; or 3) the most comprehensive measurement (e.g. total fat consumption was preferred over saturated fat). All cluster randomized studies were checked whether they accounted for clustering in their analysis. Effect estimates based upon adjustments for loss to follow-up were preferred above effect estimates of completers only. Conservative estimates were preferred, e.g. baseline observations carried forward, above random imputation of missing outcomes.

The studies varied in the use of statistics and reporting of the effect sizes. We identified six types of reported effect measures: 1) baseline and follow-up data per group; 2) data of change within each group; 3) follow-up status per group; 4) estimates of difference of change between groups; 5) numbers and fractions of participants who reached behaviour goals at follow-up; and 6) standardized effect size between groups (e.g. Cohen’s d). Whenever the data allowed, we made adjustments for baseline status. Sample size for each outcome and time-point were recorded in case of attrition or exclusion. Positive effect sizes indicated that the intervention group had a better outcome than the control group. When declining values of a measure indicated a positive effect (e.g. total fat), we reversed the effect size in order to report a beneficial intervention effect. If a study reported both physical activity and diet outcomes, we treated them as separate outcome reports in the analyses. We halved the group sizes to avoid double counting of participants and underestimating the variance associated with each effect size. Earlier studies also used this adjustment [9, 13].

### Data synthesis and analytic strategy

The results from the PA and diet trials were standardized and calculated at two time-points if available; and hereafter referred to as short and long term results. Statistical approaches were used to re-express odds ratios (from dichotomous data) as standardized mean differences allowing dichotomous and continuous data to be pooled together (Hedges’ *g* = (m_i_-m_c_)/sd_ic_). Additional file 1 describes how the overall estimate of effect was calculated as a weighted average of the intervention effects from each trial. The Stata package metan was used to produce d and SE_d_, and forest plots, and estimates of the pooled effect and heterogeneity index I^2^. It was not likely that all our included studies had the same true effect size as they used a number of different outcome measures and intervention design. Thereforee, the random-effects model was considered the most correct choice. We performed meta-analyses and compared the separate effect estimates of both diet- and PA trials at short and long term. The results were overlapping and comparable in effect size and with overlapping confidence intervals (Cis) (Table 1). We assumed that the target behaviour would not account much for the between-study heterogeneity, as previously shown in another review [9].

**Table 1:** Summary effects of behaviour change of interventions in a meta-analysis of 48 RCTs 2007-2014

Time	Short term	Long term	Short + long term
Response measure	ES 95% CI	ES 95% CI	ES 95% CI
Physical activity	0.36 (0.24,0.47)	0.25 (0.13,0.38)	0.31 (0.23,0.40)
35 trials	30 reports	17 reports	47 reports
Diet	0.41 (0.20,0.62)	0.19 (0.07,0.31)	0.29 (0.16,0.42)
26 trials	20 reports	15 reports	35 reports
PA + Diet	0.37 (0.26,0.48)	0.24 (0.15,0.33)	
61 trials	50 reports	32 reports	82 reports

*Abbreviations:* RCT: randomized controlled trial; ES: effect size; CI: confidence interval; PA: physical activity

Results from a systematic review of 48 RCTs of behaviour change interventions with ≥ 12 weeks’ duration, published from January 2007 to October 2014 for adults (mean age ≥ 40 years and with a mean BMI ≥ 30) according to type of behaviour and time of data collection (*p* < 0.0001). Short term represents outcome reports at ≤ 6 months in time, and long term represents reports at ≥ 12 months

We applied a meta-regression using the Stata-package metareg to investigate sources of heterogeneity. In this analysis, the potential predictors were bias, study characteristics and BCTs. Studies were not excluded due to high risk and/or unclear risk of bias. Instead, we explored the effects of the bias by entering each bias as independent variables in the meta-regression analyses. After checking the impact of biases with three categories, unclear and high risk of bias were merged into one category (=1) as opposed to low risk of bias (=0) with negligible alteration of results. IBM SPSS Statistics was used to record the meta-data and prepare for the meta-analyses in Stata 14. We assessed possible publication bias by visually inspecting the funnel plots from the Stata meta-bias command.

## Results

### Studies included and intervention characteristics

**Fig. 1:**
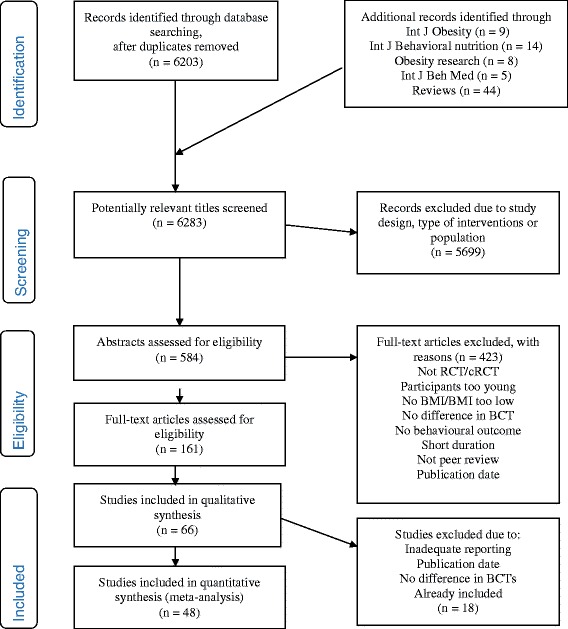
PRISMA Flow Diagram for the inclusion of studies in a systematic review of physical activity and healthy eating interventions for overweight and obese adults from January 2007 to October 2014

Forty-eight studies met our inclusion criteria and were eligible for the meta-analyses, yielding a pooled population of 11 183 participants (see Flow Chart Fig. 1 from 46 individually RCTs and two cluster RCTs [38–85]. The duration of the interventions and frequency and time of data collection varied across studies. Baseline, 6 months and 12 months were the most common time points for data collection in the 48 studies. 73% of all the interventions ended by 3 to 6 months. The duration of the interventions varied from 12 weeks to 240 weeks for PA, and from 12 weeks to 72 weeks for the diet interventions. Twenty-four studies collected data at 12 months and/or at a later time point. Twelve months was the last follow-up for 14 of these studies. Last follow-up was 240 weeks (5 years). (For the complete presentation of study and intervention characteristics see Additional files 2 and 3).

**Fig. 2:**
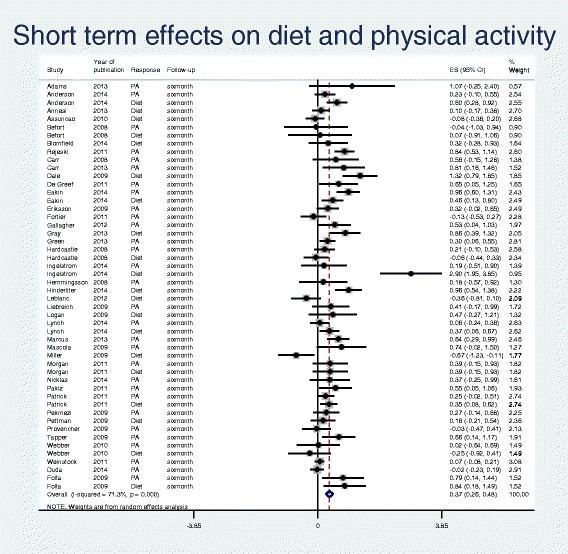
Forest plot and meta-analysis of 50 outcome reports at short term (≤ 6 months) from diet and physical activity interventions for overweight and obese adults from January 2007 to October 2014

**Fig. 3:**
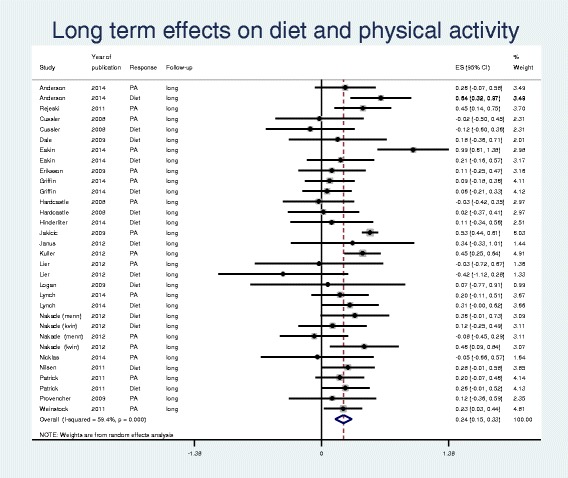
Forest plot and meta-analysis of 32 outcome reports at long term (≥ 12 months) from diet and physical activity interventions for overweight and obese adults from January 2007 to October 2014

From 48 studies, we identified 35 trials reporting PA and 26 reporting diet behaviour. These trials produced a total of 82 outcome reports for diet and PA; 50 at short term and 32 at long term (see studies and domains at short and long term, Table 1 and Figs. 2 and 3).

### Effect of physical activity and healthy eating interventions at short and long term

Table 1 reports the results from stratified meta-analyses of PA and diet outcomes at both short and long term, as well as combined. The forest plots in Figs. 2 and 3 present effect size with 95% CI for each of the outcome reports and the pooled effect sizes from short (*n* = 50) and long term (*n* = 32) reports, respectively. The estimated effect sizes were modest (0.19-0.41). The 95% CIs overlapped and showed similar effects for PA and diet, justifying pooled analyses at short and long term. It became apparent that the pooled effect size from long term (0.24) was inferior to that of short term (0.37), although the 95% CIs overlapped (0.15-0.33 and 0.26-0.48). The indexes of heterogeneity revealed strong heterogeneity for short term outcome reports (I^2^ = 71%, *p* < 0.0001) and a moderate heterogeneity for long term outcome reports (I^2^ = 59%, *p* < 0.0001).

### Bias, BCTs and other study characteristics

The Additional file 4 shows the risk of bias assessed for each of the included studies. In the eighteen studies using an objective measurement of effect, we assessed the risk for blinding of outcome assessment bias as low. This was often a PA monitoring device, e.g. an accelerometer. Most studies reported intention-to-treat analyses using “baseline observation carried forward” as a method to handle missing data from early intervention discontinuation. A few studies applied random imputation methods. High risk of attrition bias was often due to lack of information about dropouts and imbalanced attrition between the intervention- and control group. In two cases, risk of attrition bias was low at short term, but high at long term due to an unbalanced dropout. High risk of reporting bias was associated with a significant positive intervention effect at short, but not at long term, explaining 18% of the variance of results, as demonstrated in Table 2 and Additional files 6 and 7.

**Table 2:** Results from meta-regression analysis of 50 short term outcome reports of PA and diet interventions

	Simple meta-regression^a^				Multiple meta-regression^b^		
Study characteristics	b	95% CI	P value	Adj. R^2^ %	b	95% CI	*P* value
BCT 1.1 Goal setting behaviour^c^	0.480	(0.257, 0.705)	<0.001	49.2	0.440	(0.225, 0.655)	<0.001
BCT 2.2 Feedback on behaviour^c^	0.219	(−0.040, 0.479)	0.096	4.4			
BCT 2.3 Self-monitoring of behaviour^c^	0.398	(0.164, 0.632)	0.001	35.3			
BCT 2.7 Feedback on outcome of behaviour^c^	0.243	(−0.040, 0.527)	0.091	12.0			
BCT 6.1 Demonstration of the behaviour^c^	0.244	(−0.035, 0.523)	0.085	11.9			
BCT 9.2 Pros and cons^c^	−0.252	(−0.542, 0.038)	0.087	4.8			
High and unclear risk of reporting bias^d^	0.670	(0.100, 1.240)	0.022	18.5	0.530	(0.257, 1.034)	0.040
Number of BCTs unique in intervention group^e^	0.033	(0.008, 0.059)	0.012	23.8			
Source of delivery^f^							
No health professionals/unclear	0.000	reference					
Other health professionals	−0.201	(−0.550, 0.148)	0.252				
Health professionals trained in behaviour change	−0.283	(−0.607, 0.040)	0.085	6.5			
Adj. R^2^ %					58.8		

*Abbreviations and symbols: BCT* behaviour change technique, *PA* physical activity, *b* estimated meta-regression coefficient, *CI* confidence interval *Adj. R* ^*2*^ adjusted proportion of between study variance explained by predictors

^a^Simple linear meta-regression of pooled estimates of 30 physical activity and 20 diet intervention’s outcome reports. Only predictors with significant or borderline significant effects are reported; ^b^Multiple linear meta-regression: results after stepwise backwards elimination from model with all significant predictors included. Only effects with *p* < 0.05 are retained in the model. ^c^The difference of BCTs between intervention and control group contains this BCT, compared to studies not having this difference. ^d^High and unclear risk of reporting bias versus low risk; ^e^The number of unique BCTs in the intervention group as compared with the control group; ^f^Source of delivery: competence of the counsellor

When we started to code the BCTS, three researchers first coded five studies in cooperation in order to develop a joint understanding and coding practice. Thereafter GBS coded the remaining 43 studies individually whilst EM and TB individually coded 50% each. Fifty-four of 93 possible BCTs were identified as present in the intervention group, and not the control group by two researchers (see Additional file 5). Disagreement was resolved through discussions between two coders or, in two cases, by consulting the third coder. The mean kappa inter-rater agreement coefficient was 0.46 (range: 0.08 to 0.76) with an overall agreement between coders of 82% whether a BCT was present or not (range: 62 to 93%). Three of the BCTs were rated with high inter-rater reliability (>0.70) and nine reached medium interrater reliability (0.50-0.70). The remaining 17 BCTs had low interrater reliability (<0.50). In order to obtain statistical power, we included BCTs identified in a minimum of five studies in the meta-regression analyses. This left 29 BCTs for analyses. Additional files 6 and 7 presents the frequencies of the 29 BCTs, and measure of kappa and meta-regression analysis of effect.

The BCTs goal setting of behaviour and self-monitoring of behaviour were associated with positive intervention effect at both short and long term, as shown in Tables 2 and 3. Borderline significant evidence revealed that feedback on behaviour, feedback on outcome of behaviour, and demonstration of the behaviour were associated with successful interventions at short term. The BCT exploring the pros and cons of behaviour change was negatively associated (Table 2). The multiple meta-regression analyses also revealed that the BCT goal setting of behaviour and the presence of reporting bias significantly predicted between-study variation, explaining 58.8%. However, strong inter-correlation existed between goal setting of behaviour (BCT 1.1) and self-monitoring of behaviour (BCT 2.3) (Chi squared = 33, df = 1). Therefore, we substituted BCT 1.1 with 2.3, in the final step of the regression analysis. Self-monitoring of behaviour was also significantly associated with intervention effect (b = 0.355; 95% CI: 0.128 to 0.582), but this model only explained 46.7% of the variance.

**Table 3:** Results from meta-regression analysis of 32 long term outcome reports of PA and diet interventions

	Simple meta-regression^a^				Multiple meta-regression^b^		
Study characteristics	b	95% CI	P value	Adj. R^2^ %	b	95% CI	*P* value
BCT 1.1 Goal setting behaviour^c^	0.228	(0.056, 0.400)	0.011	38.5	0.175	0.043, 0.307	0.011
BCT 1.2 Problem solving^c^	0.161	(−0.005, 0.327)	0.057	25.1			
BCT 1.3 Goal setting outcome^c^	0.256	(0.095, 0.416)	0.003	53.2			
BCT 1.5 Review behaviour goals^c^	−0.319	(−0.678, 0.040)	0.078	19.8			
BCT 2.3 Self-monitoring of behaviour^c^	0.184	(0.009, 0.360)	0.040	30.8			
BCT 2.7 Feedback on outcome of behaviour^c^	0.249	(0.085, 0.412)	0.004	43.8	0.145	0.021, 0.269	0.024
BCT 3.1 Social support (unspecified)^c^	0.192	(−0.011, 0.394)	0.063	21.6			
BCT 8.7 Graded tasks^c^	0.203	(0.043, 0.363)	0.014	37.1			
BCT 12.5 Adding objects to the environment^c^	0.182	(0.010, 0.354)	0.039	12.7			
Method based^d^							
MI/SDT	0.000	reference					
ACT/CT/HAES/Mindful/other	−0.303	(−0.500, −0.105)	0.004				
Unclear	−0.199	(−0.372, −0.026)	0.026	57.5	−0.170	−0.294, −0.045^g^	0.009
Number of BCTs unique to the intervention group^e^	0.028	(0.012, 0.044)	0.001	54.3			
Total number of BCTs in intervention + control group^**f**^	0.030	(0.014, 0.046)	0.001	61.3			
Adj. R^2^ %					100.0		

*Abbreviations and symbols: BCT* Behaviour change technique, *PA* physical activity, *ß* estimated meta-regression coefficient, *CI* confidence interval, *Adj. R* ^*2*^ adjusted proportion of between study variance explained by predictors

^a^Simple linear meta-regression of pooled estimates of 17 physical activity and 15 diet intervention’s outcome reports. Only predictors with significant or borderline significant effects are reported; ^b^Multiple linear meta-regression: results after stepwise backwards elimination from model with all significant predictors included. Only effects with *p* < 0.05 are retained in the model; ^c^The difference of BCTs between intervention and control group contains this BCT, compared to studies not having this difference. ^d^Method-based interventions comparing MI or SDT based interventions with Acceptance and commitment therapy (ACT), Cognitive therapy (CT), Health-at-every-size (HAES) approach, Mindful based intervention or other methods, versus no method mentioned; ^e^The number of unique BCTs in the intervention groups as compared with the control group; ^f^The total number of BCTs in intervention and control group; ^g^The variable is dichotomized in the multiple meta-regression analysis to MI/SDT versus all others

In addition to the BCTs goal setting and self-monitoring of behaviour, giving feedback on the outcome of behaviour, setting graded task, and adding objects to the environment, e.g. using a diet logbook, were associated with successful intervention reports at long term. As Table 3 demonstrate the BCTs problem solving, review of behaviour goals, and receiving general social support, were borderline significantly associated with positive results. In addition to the effect of using different BCTs, the multiple stepwise meta-regression analysis revealed that three study characteristics had independent explanatory power. Applying an autonomy supportive communication style in counselling, e.g. MI and SDT based interventions, the BCTs goalsetting of behaviour and receiving feedback on the outcome of behaviour, were all associated with trial effects, explaining 100% of the between study variation. Strong inter-correlation existed between feedback on outcome of behaviour (BCT 2.7) and goalsetting of outcome (BCT 1.3) (Chi squared = 30, df = 1). Therefore, we substituted both BCT 1.1 with 2.3 and BCT 2.7 with BCT 1.3 in the final step of the regression analyses. Goalsetting of outcome (BCT 1.3) was significantly associated with outcome effect (b = 0.149; 95% CI: 0.005 to 0.292), whereas self-monitoring of behaviour (BCT 2.3) only reached borderline significance (p = 0.059). This model still predicted 100% of the variance.

In the Introduction, we argued that SDT based interventions often use MI as a person-centred communication style to promote internal and autonomous motivation for change. However, when we compared all theory-or model-based trials with other trials, we found no evidence, neither at short or long term, that theory-based interventions were associated with between study effects. We did not identify any associations between treatment effects and 1) using objective versus self-reported data; 2) being in a community or workplace setting versus primary care or hospital; 3) receiving an individual or group based intervention; and 4) promoting behaviour change in one domain versus two (both diet and PA).

### Publication bias

We assessed publication bias by inspection of funnel plots, see Additional files 8 and 9. The funnel plot of short term reports showed a fairly symmetrical distribution, demonstrating low risk of publication bias. The funnel plot of long term reports was asymmetrical, and revealed an over-representation of publications of small studies with low effects.

## Discussion

### Main results

The present review shows that behaviour change interventions for diet and PA are modestly effective both at short and long term, and that the heterogeneity between studies is high, especially at short term. However, we have revealed study characteristics that explain most of the variance between studies. In particular, several BCTs that facilitate self-regulation of behaviour explain intervention effects, e.g. the BCTs goalsetting of behaviour and self-monitoring of behaviour. Interventions that emphasize a person-centred and autonomy supportive communication style, as MI, SDT and other autonomous based interventions, are associated with effects at long term. Facilitating self-regulation and sustained positive motivation are previously identified as two important themes in theoretical explanations for maintenance of behaviour change [5].

### Strengths and limitations

In the present review, we have applied an internationally validated taxonomy identifying BCTs [6]. Two researchers coded risk of bias and BCTs independently and came to an agreement through discussion. We included only RCTs and adjusted for baseline status whenever possible. By applying a search strategy formerly used with high utility [10], we maintain that a comprehensive collection of relevant papers was found. We have complied with a predefined protocol published at the start of the study. Statistical methods were in line with formerly advocated methods [9]. We also checked for correlations of BCTs, a previous methodological weakness pointed out by Peters and colleagues [35]. Unlike previous reviews, we have collected outcome reports at two points in time in order to differentiate between short and long term intervention effects. However, we do acknowledge that 12 months is a rather short timeframe for evaluating long term maintenance.

Modest inter-rater reliability was obtained in coding despite completing an online education and certification. The descriptions of the interventions’ BCTs and other study characteristics were at times limited and lacked precision, even after checking the protocol article. Only a minority of the studies reported the fidelity. We do not know to what extent reported interventions were implemented as planned. The results of this review are also limited by the fact that the inclusion of RCTs stopped in October 2014. The methodological procedures, involving several researchers, have been thorough and time consuming. We have updated our search once but a second update proved impossible due to time restrictions.

### Our findings compared with other studies

Our pooled effect estimation of interventions for PA at short term are comparable to some previous reviews [9, 86], higher than one [11, 12], and lower than another [87]. Our pooled effect for diet interventions was lower than in one comparable study [11]. As far as we are aware no reviews using the BCTTv1 [86, 88, 89] have performed meta-analyses combining healthy eating and PA interventions among overweight and obese adults, and used meta-regression to examine differences in effect size as a function of BCTs or other study characteristics. Previous reviews have used either the 26 or the 44 BCT taxonomy [8, 9], on various target populations, behaviours, and used different meta-analytic strategies. Unlike these, we only recorded BCTs present in the intervention and absent in the control condition. Therefore, our ability to compare our findings with former studies was somewhat limited.

However, results from this study showed that helping participants to define a goal, e.g. eating five fruit and vegetables per day, or to monitor the behaviour, for instance in a log book, were independently associated with better intervention effects. These results are supported by earlier studies for the BCT goalsetting of behaviour [13, 89], and self-monitoring of behaviour [9, 10, 90]. Our analyses suggest that these BCTs also affected long term results. As expected, having more BCTs unique to the intervention group, and not the control group, were associated with larger effect sizes at both short and long term. A previous study have illustrated how the content of the control condition, e.g. waiting list, usual care or alternative treatment may influence the effect size [86]. Using BCTs that help the participant to identify realistic outcomes of a new behaviour, e.g. reduce CVD risk factors, or when counsellors give feedback on results, e.g. praising efforts, were independently associated with intervention effect at long term. The effect of outcome feedback has also been reported by Lara and colleagues [13], and contrasted in another study which demonstrated a negative effect [11]. Applying the BCTs setting graded tasks and adding objects to the environment, e.g. using a mobile app to register PA, were independently associated with intervention success at long term. As far as we know, no previous reviews which used any of the taxonomies [6–8] have associated these BCTs with intervention effects, except one study which reported a negative impact of using graded tasks [90].

Using the BCTs problem solving (e.g. to identify barriers or facilitators for change), review of behaviour goals, and receiving social support (e.g. from staff or other participants) were borderline significantly associated with positive outcomes at long term. Problem solving and planning of social support have previously been associated with effects in diet and smoking cessation counselling [13, 91]. Theoretical explanations and self-regulation models for behaviour change maintenance recommend the use of these BCTs [5, 92]. The BCT to explore the pros and cons argument of change during the intervention were borderline significant and negatively associated with the intervention effect. This is not surprising. Exploring ambivalence may improve motivation among people not ready for behaviour change, but can actually hamper motivation when the client is ready for change. In these cases a more action oriented counselling seems more beneficial [93].

In line with earlier studies [16, 88], we found no evidence that the mode of intervention delivery was associated with intervention effects. This finding supports the notion that a wide range of providers can deliver effective diet and physical activity interventions, both professionals and lay people. Unlike previous findings we found no effect of treatment settings [10]. Increasing the number of total BCTs was associated with positive intervention results as also confirmed by other studies [13, 86].

There were no evidence, neither at short term nor at long term, that theory-based interventions were associated with positive results. It was beyond the scope of this review to consider if and how the theory was applied in the intervention design, e.g. if theory relevant constructs or predictors were linked to intervention techniques [15, 94]. Unlike Wilson and colleagues we did not identify any associations between promoting behaviour change in one domain versus two (diet plus PA) and trial effects [95].

### Behaviour change initiation and maintenance

Meta-regression analyses revealed that person-centred methods as in Motivational Interviewing, SDT and other autonomous supporting interventions were associated with maintenance of change at ≥12 months. Previously, only a few PA interventions have reported positive intervention effect at more than 12 months [16, 30, 96]. Dietary interventions have showed positive changes at 6 to 19 months [16]. Our findings suggest that setting a goal for behaviour change and to monitor the new behaviour are effective in helping people to both initiate change and to maintain the change. In line with theoretical explanation of maintenance, the focus will change from expectations of future outcomes to experiences with results over time; the cost and limitation of self-regulation, setbacks, and relapses [5]. BCTs like goalsetting of outcome, setting graded tasks, and getting feedback on outcome, highlights the results achieved and the possible satisfaction with the new behaviour. If counselling is performed in a person centred and autonomous supporting manner, the participants’ self-regulatory strength may be renewed by developing a genuine appreciation of healthy food, and development of autonomy (sense of choice, feeling volitionally), and internalization of the new behaviour into the person’s perceived values, aspirations, and autonomous self-regulations [31].

The results from the present review supports two theoretical themes important in maintenance of change [4, 5]; BCTs facilitating behaviour self-regulation, e.g. skills and functional aspects of behaviours (“how to”), combined with a communication style that addresses the underlying nature of motivation (“the why”) in order to maintain the new behaviour over time. These perspectives are not opposites, but complement each other. Without the first, there would be lack of competence. Without the second, there is lack of meaning, value, and satisfaction of psychological needs.

### Can BCT research inform counselling practice?

Efforts to identify effective BCTs using taxonomies have been criticized for ignoring the manner by which the BCTs are presented. Hagger and colleagues argue that the interpersonal style represents a unique set of techniques and should be included in the taxonomies [97]. When coding the MI, SDT or ACT based interventions for this review we experienced a lack of relevant techniques, and we were unable to code e.g. eliciting the “promoting participants own reasons for change”; “unconditional personal regard”; “provision of choice” and; “in an autonomy supportive manner”. Additionally, in this review we had to exclude one study because it was impossible to code the difference in “restrictive” and “positive” messages in counselling, although we felt that this was a rather important difference [98]. We should also acknowledge Jane Ogden’s warnings that the promotion of BCTs as strict techniques may hamper professional variability and turn professionals into technicians [99]. The present study supports the importance of applying the techniques with professional respect and empathy.

## Conclusions

There are similarities, but also differences in effective BCTs promoting change in healthy eating and physical activity and BCTs supporting maintenance of change. The results support the use of goal setting and self-monitoring of behaviour when counselling overweight and obese adults. Several other BCTs as well as the use of a person-centred and autonomy supportive counselling approach seem important in order to maintain behaviour over time.

## Additional files

Additional file 1: Computation of standardized mean differences (DOCX 14 kb)

Additional file 2: 48 physical activity and diet studies included in review (DOCX 39 kb)

Additional file 3: Intervention characteristics of 48 PA and diet studies included in review (DOCX 44 kb)

Additional file 4: Risk of bias in 48 included studies by first author (DOCX 32 kb)

Additional file 5: BCTs unique to the intervention and not in the control group coded by The behaviour change technique taxonomy. (DOCX 29 kb)

Additional file 6: Table 4. Results from simple linear meta-regression analysis of short term reports of PA and diet interventions. (DOCX 35 kb)

Additional file 7: Table 5. Results from simple linear meta-regression analysis of long term reports of PA and diet interventions. (DOCX 33 kb)

Additional file 8: Figure 4. Funnel plot short term. (DOCX 15 kb)

Additional file 9: Figure 5. Funnel plot long term. (DOCX 15 kb)

## Abbreviations

BCT: Behaviour change technique
CI: Confidence interval
CT: Cognitive therapy
ES: Effect size
HAES: Health-at-every-size
MI: Motivational interviewing
NCD: Non-communicable disease
PA: Physical activity
RCT: Randomized controlled trial
SDT: Self-determination theory
se: standard error
SMD: Standardized mean difference
