# Supplementary material for: Effective behaviour change techniques for physical activity and healthy eating in overweight and obese adults; systematic review and meta-regression analyses
Source: Int J Behav Nutr Phys Act. 2017 Mar 28;14:42. doi: 10.1186/s12966-017-0494-y (PMC5370453; doi:10.1186/s12966-017-0494-y)
Supplement: Supplementary file 1 — Computation of standardized mean differences (DOCX 14 kb) [file 12966_2017_494_MOESM1_ESM.docx]

# Additional file 1 Computation of standardized mean differences

The overall estimate of effect was calculated as a weighted average of the intervention effects from each trial using statistical methods as follows:

Let M denote the mean, t the time of measurement (0 = baseline), and i the treatment group (i = 1, 2 for control and treatment, respectively). According to the six types of effect measures reported the following effect measures were recorded for i = 1, 2:

1) With M_ti_, S_ti_, and n_ti_ at t = 0 and at some t > 0 the mean differences D_ti_ = M_ti_ - M_0i_ could be calculated, but the standard deviation of the difference could not be. Thus the estimates at t > 0 were chosen and recorded, i.e. M_ti_, S_ti_ and n_ti_; 2) Here the mean changes until t > 0, D_ti_ , and the standard deviations S_ti_ for the changes were available directly and recorded; 3) In this case M_ti_, S_ti_ and n_ti_ at time t > 0 were recorded directly; 4) If D denotes the estimate of difference of change between the two groups, we estimated the standardized mean difference, d, and its standard deviation, SE_d_, using the theory in Borenstein and collegues [1], i.e. by formulae (4.18) and (4.20) [1]:

$d = \frac{D}{S_{within}}$ with $S_{within}=S_{pooled}=\sqrt{\frac{n_{1}n_{2}}{n}}{SE}_{D}$

and SE_D_ = √V_D_ with $V_{D}= \frac{n_{1}+n_{2}}{n_{1}n_{2}}+\frac{d^{2}}{2(n_{1}+n_{2})}$

5) With the count of individuals that obtained their goal at time t > 0, also denoted by M_it_, these were recorded and their estimated standard deviations calculated as S_it_ = √[M_ti_(n_ti_-M_ti_)/n_ti_] and recorded; 6) The standardized effect size and its standard deviation, d and SE_d_, was recorded directly.

1. Borenstein M, Hedges LV, Higgins J, Rothstein HR: *Front matter.* Wiley Online Library; 2009.
