# Supplementary material for: Effective behaviour change techniques for physical activity and healthy eating in overweight and obese adults; systematic review and meta-regression analyses
Source: Int J Behav Nutr Phys Act. 2017 Mar 28;14:42. doi: 10.1186/s12966-017-0494-y (PMC5370453; doi:10.1186/s12966-017-0494-y)
Supplement: Supplementary file 2 — 48 physical activity and diet studies included in review (DOCX 39 kb) [file 12966_2017_494_MOESM2_ESM.docx]

| **First author &**  **Publication year** | **Design**  **Name of study** | **Participant information**  **Health status**  **Mean age**  **Mean BMI**  **Gender (%female)**  **Ethnicity** | **Intervention**  **+**  **Control group**  **sample size** | **Total retention rate at end of study** | **Time points data collection and last follow-up** | **Outcomes collected** | **Outcome(s) included in review**  **(S) = Self-report**  **(O) = Objective**  **PA= Physical activity** |
| --- | --- | --- | --- | --- | --- | --- | --- |
| Adams 2013 | RCT | Inactive overweight  Age 37  BMI 30  85% female  35 % non -white | Int N 10  Con N 10 | 90 % | 0, 6 months  =24 weeks | Steps/day | PA: Steps/day (O) |
| Anderson 2014 | RCT | Overweight/obese  Age 64  BMI 31  74% men  100 % white | Int N 163  Con N 166 | 93% | 0,6,12 months  = 48 weeks | Weight, waist circumference, blood pressure, fasting cardiovascular biomarkers, glucose metabolism variables, physical activity, diet, alcohol consumption | PA: Steps/day (O)  Diet: Fat % (S) |
| Annesi 2013 | RCT | Sedentary adults  Age 44 years  BMI 39,8  81% female  52% White  46% Black  2 % Other | Int N 101  Con N 99 | 86% | 0,3,6 months  = 24 weeks | Exercise, fruit and vegetable intake  self-regulation, self-efficacy, mood | Diet: Fruit &Vegetable (S) |
| Assuncao 2010 | RCT | Overweight/obese  Age 40  BMI 34  89 % female | Int N 120  Con N 121 | 80% | 0, 6 months  = 24 weeks | Weight, BP, lipids and glucose levels, physical activity, diet | Diet: Fats (S) |
| Befort 2008 | RCT | Obese African American women  Age 44  BMI 40 | Int N 21  Con N 23 | 77% | 0,16 weeks | Weight loss, dietary outcome, physical activity, motivation, self-efficacy | PA: Min /week (S)  Diet: Energy intake kcal /day (S) |
| Blomfield 2014 | RCT | Overweight/ obese  Age 47  BMI 33  100% men | Int N 53  Con N 52 | 81% | 0,3,6 months  = 24 weeks | Weight, dietary intake, portion size | Diet: Fat/day (S) |
| Rejeski 2011 | RCT | Overweight/ obese with CVD or risk for CVD  Age 67  BMI 33  66% Female  82 % White  17 % Black  1 % Other | Int N 98  Con 93 | 86% | 0,6, 18 months  = 72 weeks | 400-m walk,, weight loss, level of physical activity, adverse events | PA: Moderate to vigorous /week (O) |
| Carr 2008 | RCT | Sedentary, overweight/ obese  Men & women  Age 35  BMI 31 | Int N 14  Con N 18 | 51% | 0,16 weeks | Physical activity, fitness, anthropometric and body composition, metabolic measures | PA: Steps /day (O) |
| Carr 2013 | RCT  Pedal@Work | Sedentary/ overweight  Age 45  BMI 32  90% Female  70% Non-Hispanic White | Int N 25  Con N 24 | 81% | 0,12 weeks | Sedentary and physical activity, heart rate, blood pressure, height, weight, waist circumference, per cent body fat, cardiorespiratory fitness, fasting lipids | PA: Sedentary time/day (O) |
| Cussler 2008 | RCT | Females after a 4-month weight loss treatment  Age 48  BMI 31 | Int N 66  Con N 69 | 82% | 0, 12 months  = 48 weeks | Weight loss maintenance, anthropometric and body composition, physical activity, diet | PA: Exercise energy expenditure (kcal/day) (S)  Diet: Energy intake (kcal/day) (S) |
| Dale 2009 | RCT | Overweight insulin resistant  Age 46  BMI 34  67% female  100 % Caucasian | Int N 56  Con N 23 | 78,5% | 0,4,8,12,24 months  = 96 weeks | Weight loss, anthropometric and body composition , blood pressure, fasting glucose, lipids, insulin and aerobic fitness, dietary intake | Diet: Total fat (S) |
| De Greef 2011 | RCT | Diabetes type 2  Age 67  BMI 30  30 % Female | Int 1 N 21  Con N 67 | 96% | 0,12 weeks | Pedometer-determined steps/day, physical activity , weight, body mass index, waist circumference, total cholesterol, fasting glucose, HbA1c | PA: Steps/day (O) |
| Eakin 2014 | RCT | Diabetes type 2  Age 58  BMI 33  44% Female  68% born in Australia  87 % Caucasian | Int N151  Con N151 | 77% | 0,6,18,24 months  =96 weeks | Weight, physical activity, HbA1c,  dietary energy intake, diet quality, waist circumference, lipid levels, blood pressure | PA: Moderate to vigorous /week (O)  Diet: quality (S) |
| Eriksson 2009 | RCT  The Swedish Bjørknes study | Risk of CVD  Age 54  BMI 30  57% Female | Int N71  Con N 74 | 80 % | 0,12,24,36 months  =144 weeks | Anthropometrics, aerobic fitness, self-reported physical activity, blood pressure, metabolic traits | PA Total (S) |
| Fortier 2011 | RCT  The physical activity Counselling (PAC) trial | Sedentary  Age 47  BMI 31  69 % Female  99 % Caucasian | Int N 61  Con N 59 | 82% | 0,6,12,18,  25 weeks | Physical activity, quality of life, metabolic outcomes | PA: Activity counts per minutes (O) |
| Gallagher 2012 | RCT  Healthy Eating and Exercise Lifestyle Program | Overweight or obese with CHD and/or Diabetes type 2  58% Male  Age 63  BMI 31  79 % Caucasian | Int 83  Con 65 | 91% | 0,12 weeks + 6, 12 months  = 48 weeks | Weight loss, waist circumference and exercise, self-efficacy | PA:  Duration, min/week (S) |
| Gray 2013 | RCT | Overweight/obese men  Age 47  BMI 35 | Int N 51  Con N 52 | 84% | 3, 6, 12 months  = 48 weeks | Weight, waist circumference, blood pressure, body composition, physical activity, diet, alcohol consumption, psychological outcomes, qualitative data | Diet: Fruit & vegetable (S) |
| Greene 2013 | RCT | Overweight/obese  78 % over 40 years’ old  93 % overweight/obese  79% Female  93% Caucasian  7% Other | Int N 137  Con N 125 | 68% | 0,3,6 months  = 24 weeks | Physical activity, weight, cholesterol, and triglycerides | PA: min/week (O) |
| Griffin 2014 | The ADDITION-Plus RCT | BMI 32  Age 59  62 % men  97% White | Int N 239  Con N 239 | 94% | 0,12 month  =48 weeks | Physical activity energy expenditure, fruit and vegetable intake, medication adherence, smoking status | Diet:  C vitamin plasma (0)  PA: Energy expenditure kJ/day (O) |
| Hardcastle  2008 | RCT | Overweight/ obese  BMI 34  Age 51  67 % Female | Int N 203  Con N 131 | 65% | 0.6, 18 months  =72 weeks | Diet, physical activity, body mass index, weight, blood pressure and cholesterol | Total PA (S)  Diet: Fruit & vegetable (S) |
| Ingelstr$ö$m 2014 | RCT | Obstructive sleep  apnea syndrome and overweight  Age 55  BMI 35  20 % Female | Int 36  Con 30 | 72% | 0,6 months  =24 weeks | Diet, eating behavior, weight, BMI, waist circumference, PA, sedentary time  . | PA: Steps (O)  Diet: Vegetable /week (S) |
| Hemmingsson 2008 | RCT | Obese  Age 43  BMI 42  79 % Female | Int N 28  Con N 27 | 76% | 0,18 weeks | Steps /day | PA Steps /day (O) |
| Hinderliter 2014 | RCT | Overweight with high BP  Age 52  BMI 33  67 % Female  60 % White  39 % Black  1% Asian  3 % Hispanic | Int N 49  Con N 49 | 86% | 0, 4, 12 months  =48 weeks | BP, weight, dietary intake, exercise habits, medication | Diet: Total kcal/day (S) |
| Jakicic 2009 | RCT  The Look AHEAD trial | Diabetes type 2  Age 58  BMI 36  59% Female  36% Non-Caucasian | Int N 1118  Con N 1103 | 85% | 0, 12 month  = 48 weeks | Fitness, physical activity | PA: Leisure-time kcal/week (S) |
| Janus 2012 | RCT | Risk of Diabetes type 2  Age 65  BMI 31  66% Female | Int N 49  Con N 42 | 87% | 0, 12 months  = 48 weeks | Anthropometric, laboratory tests, psychosocial, diet, physical activity  depression and anxiety | Diet: Total fat % (S) |
| Kuller 2012 | RCT  The Women on the Move through Activity and Nutrition (WOMAN) study | Women at risk for CVD  Age 57  BMI 31  88% Caucasian | Int N 253  Con N 255 | 90% | 0, 18,36,48 months  =192 weeks | Laboratory, subclinical and anthropometric measurements, drug treatment, weight loss, diet, physical activity | PA: Leisure time MET h/week (S) |
| Leblanc 2012 | RCT | Overweight/ obese women  Age 42  BMI 36 | Int N 48  Con N 46 | 87,7% | 0,4 6 months  =24 weeks | Anthropometric measurements, dietary intakes, eating patterns | Diet: Energy intake (kcal) (S) |
| Liebreich 2009 | RCT | Diabetes type 2  Age 54  BMI 34  59% Female | Int N 25  Con N 24 | 90% | 0,12 weeks | Physical activity, several social cognitive measures, e.g. self-efficacy, self-regulation, observational learning, social support | PA: Moderate to vigorous min /week (S) |
| Lier 2012 | RCT | Obese (Bariatric surgery)  Age 42  BMI 45  73% Female | Int N 49  Con N 50 | 78% | 0, 12 months  = 48 weeks | Anthropometric measurements, diet, anxiety, depression, quality of life | PA: Min/week (S)  Diet: Meals each day (S) |
| Logan 2010 | RCT | Coronary heart disease  Age 57  BMI 30  20 % Female | Int N 19  Con N 18 | 60% | 0,6,12 months  =48 weeks | Demographic variables, diet, smoking status, weight, height, blood pressure and sample | Diet: Mediterranean diet score (S) |
| Lynch 2014 | RCT | Survivors of colorectal cancer  Age 66  BMI insufficiently reported  46% Female | Int N 205  Con N 205 | 79% | 0, 6, 12 months  =48 weeks | Demographic variables, anthropometric measurements, diet, physical activity, cancer-related fatigue, health- related quality of life | PA: Total sedentary time h/day (S)  Diet: Total fat % of kJ intake (S) |
| Marcus 2013 | RCT  The Seamos Saludables Study | Inactive Latinas  Age 40  BMI 30  100 % Female | Int N 132  Con N 134 | 86% | 0,6,12 months  =48 weeks | Physical activity | PA: Moderate to vigorous min /week (S) |
| Mascola 2009 | RCT | Overweight/obese  Age 43  BMI 35  92% Female  77% White | Int N 14  Con N 12 | ? | 0,3 months  =12 weeks | Physical activity/week, cardiorespiratory fitness, and depression | PA: Hours/week of moderate-equivalent (S) |
| Miller 2009 | RCT | Diabetes type 2  Age 54  Weight mean 106 kg  64 % Female  77% Caucasian | Int 32  Con 36 | 76% | 0,3 months  =12 weeks | Weight, anxiety/depression, outcome expectations, nutrition and eating-related self-efficacy, eating behaviors (cognitive control, disinhibition, hunger), mindfulness | Diet: Energy (kcal) (S) |
| Morgan 2011 | Cluster RCT  The Workplace POWER (Preventing Obesity Without Eating like a Rabbit) | Male shift workers  Age 44  BMI 30 | Int N 65  Con N 45 | 81% | 0,14 weeks | Weight, waist circumference, BMI, blood pressure, resting heart rate, physical activity, diet, physical activity and dietary cognitions | PA: Total MET minutes (S)  Diet: Fruit servings/day (S) |
| Nakade 2012 | RCT  Saku Control Obesity Program (SCOP) | Overweight/obese  Age 54  BMI 30  50 % Female | Int N 119  Con N 116 | 96% | 0,12,24 months  =96 weeks | Anthropometric and biological data, physical activity, diet and eating behavior, stages of change | Diet: Energy kcal/day (S)  PA steps/day (O) |
| Nicklas 2014 | RCT | Obese  Age 70  BMI 33  76% Female  86% Non-Hispanic white  14 % Non-white | Int N 23  Con N 23 | 85% | 0, 5,10 months  =40 weeks | Body weight, physical activity, energy expenditure | PA: Steps/day (O) |
| Nilsen 2011 | RCT | Risk of diabetes type 2  Age 47  BMI 37  50% Female | Int N 109  Con N 104 | 85% | 0,3, 26 weeks, 18 months  =72 weeks | Anthropometric and biological data, blood sample, diet quality, physical test | Diet: Smart Diet Score (S) |
| Pakiz 2011 | RCT | Overweight breast cancer survivors  Age 56  BMI 31  94% Non-Hispanic White | Int N 44  Con N 24 | 81% | 0,16,48 weeks | Anthropometric and biological data, physical activity | PA: PA recall (S) |
| Patrick 2011 | RCT | Overweight/ obese  Men  Age 44  BMI 34  71% White non-Hispanic  18% Hispanic  12 % Others | Int N 224  Con N 217 | 70% | 0,6,12 months  =48 weeks | Anthropometric data, BMI, diet, physical activity | PA. Walking min/day (S)  Diet: Fruit & vegetable servings/day (S) |
| Pekmezi 2009 | RCT  Seamos Activas | Overweight/obese Latino/Hispanic women  Age 41  BMI 47 % obese | Int N 45  Con N 48 | 87% | 0,6 months  =24 weeks | Physical activity, cognitive and behavioral processes of change, self-efficacy, social support, depression, access to nearby physical activity facility | PA: Moderate physical activity (S) |
| Pettman 2009 | RCT | Metabolic syndrome  Age 45  BMI 37  73% Female | Int N 103  Con N 50 | 91% | 0,4 months  =16 weeks | Anthropometric, physical fitness, diet, body composition, cardio-metabolic risk factors, fasting blood sample | Diet: KJ reduction (S) |
| Provencher 2009 | RCT | Premenopausal overweight/obese women  Age 42  BMI 30  93% White | Int N 48  Con N 48 | 74% | 0,4,6 12 months  =48 weeks | Eating behaviors, appetite sensations, metabolic and anthropometric variables, and physical activity levels | PA mean energy exp (S) |
| Tapper 2009 | RCT | 100 % Women  Age 41  BMI 32 | Int N 31  Con N 31 | 81 % | 0,4,6 months  =24 weeks | BMI, physical activity, mental health | PA: Bouts of 30 m /week (S) |
| Webber 2010 | RCT | 100 % Female  Age 49  BMI 32  92% Caucasian | Int N 40  Con N 40 | 82% | 0,4,16 months  =64 weeks | Body composition , controlled and autonomous motivation, diet, PA, weight loss self-efficacy, depression | PA: Increase in PA (S)  Diet: Decrease energy intake kcal/day (S) |
| Weinstock 2011 | RCT  IDEATel study | Ethnically diverse, medically underserved Medicare beneficiaries with diabetes  63% Female  Age 71  BMI 32  White 50%  Black 15%  Hispanic 35% | Int N 837  Con N 813 |  | 0,3,6 months, 5 years  =240 weeks | Hemoglobin A1c, social support, decline in PA and physical impairment, comorbidity, activities of daily living, diabetes self-care activities | PA: Diabetes self-care PA score (S) |
| Duda 2014 | Cluster RCT | 90% overweight /obese  Age 89% over 30  72,9 % Female  28% Non-white | Int N 184  Con N 163 | 56% | 0,3,6 months  =24 weeks | Physical activity, health status, emotional well-being, anxiety, depression, quality of life, vitality, autonomy support, need satisfaction, intentions to be active, motivational regulations for exercise, BP, weight | PA: Moderate -vigorous min/week (S) |
| Folta 2009 | Cluster RCT Strong Women–Healthy Hearts intervention | Sedentary midlife and older overweight/obese  100% Female  Age 57  BMI 33 | Int N 61  Con N 35 | 89% | 0,12 weeks | Anthropometric data, diet, PA | PA: Steps (O)  Diet: Energy intake kcal/day (S) |

*Abbreviations:* RCT = randomized controlled trial. Int = Intervention grou. Con = Control group. BMI = Body Mass Index. PA= physical activity. S= Self-reported data. O= Objective measure.
