# Supplementary material for: Effective behaviour change techniques for physical activity and healthy eating in overweight and obese adults; systematic review and meta-regression analyses
Source: Int J Behav Nutr Phys Act. 2017 Mar 28;14:42. doi: 10.1186/s12966-017-0494-y (PMC5370453; doi:10.1186/s12966-017-0494-y)
Supplement: Supplementary file 3 — Intervention characteristics of 48 PA and diet studies included in review (DOCX 44 kb) [file 12966_2017_494_MOESM3_ESM.docx]

|  | | | | | | |
| --- | --- | --- | --- | --- | --- | --- |
| **First author of study** | **Intervention group** | **Control group** | **Source of delivery**  **Treatment setting** | **Duration for intervention**  **Intensity** | **Format** | **Theory mentioned in abstract, introduction or method section** |
| Adams 2013 | An adaptive physical activity intervention | A static physical activity intervention | Unclear  Community, USA | 24 weeks  Email and text message communication every day  and biweekly motivational prompts | Website  Individual |  |
| Anderson 2014 | BeWEL programme | A weight loss booklet | Trained lifestyle counsellors  Cancer screening clinics, NHS Scotland | 48 weeks  three, one hour, face to face visits with a lifestyle counsellor during the first three months (including spouse or friend when possible), followed by nine, monthly, 15 minute telephone consultations | Face to face  Individual  Telephone |  |
| Assunco 2010 | Nutritional care (dietetic prescription) | Usual care (individual consultations with nutritionist in public health system) | Nutritionists  Nutrition Outpatient Clinic, Medical Teaching Hospital, Brazil | 24 weeks  Individualized nutritional care | Face to face  Individual |  |
| Anessi 2013 | Cogn. behavioural exercise support + Cogn.beh. methods for nutrition change | Cogn.behavioural exercise support + stand. nutrition education | Certified YMCA wellness leaders  Community, USA | 24 weeks  Individual exercise support 6 x 1 hour meetings  Group based nutritional component 6 x1 hour meetings | Face to face  Group  Individual | Self-efficacy Theory  Social Cognitive theory  Theory of Self-regulation |
| Befort 2008 | Behavioral weight loss program plus MI sessions | The same behavioral weight loss program plus four health education sessions | A doctorate-level psychologist  and a masters-level counsellor or dietitian trained in MI  Community health center  Serving, USA | 16 weeks  90-min weekly group sessions plus 4 x 30-min  MI sessions | Face to face  Group  Telephone |  |
| Blomfield 2014 | Information resources with access to a website for self-monitoring of diet and exercise with individualized e-feedback | Waiting list | Research assistants using a standardized set of feedback sheets  Community, Australia | 12 weeks  Email and 7 indiv. feedback sheets. Diaries reviewed weekly in the 1st month, fortnightly in the 2nd month and once in the 3rd month. DVD, Handbook, Support book | Website  Individual | Social Cognitive Theory |
| Rejeski 2011 | A physical activity program in conjunction with dietary weight loss | Successful aging education control | Cooperative Extension agents  Community, USA | 72 weeks  Months 1 to 6 Intensive phase: 3x 90 minutes’ group sessions and 1x 30 minutes’ individual session per month. Months 7 to 18 Maintenance phase: 2 contacts each month (group session, telephone contact) | Face to face  Group  Individual  Telephone | Social Cognitive Theory |
| Carr 2008 | Internet-delivery of the Active Living Every Day (ALED program and workbook) | Maintain current behaviour | Licensed program administrators/  facilitators  Community, USA | 16-week  A self-paced program using of interactive activities. Journal activity | Website  Individual | Transtheoretical Model of Behavioural Change  Social Cognitive Theory |
| Carr 2013 | An intervention to reduce sedentary time | Maintain current behaviour | Unclear  University worksite, USA | 12 weeks  A pedal machine connected to the PC during all working hours. Daily email messages to self-monitor, daily pedal time and daily steps on the website. Virtual competition for groups of intervention participants. Three motivational emails each week. | Website  Individual  Email | Social Cognitive Theory |
| Cussler 2008 | A weight maintenance internet intervention | No contact (self-directed weight maintenance) | Study staff  Community, Arizona area, USA | 48 weeks  Progress monitoring tools (body weight, physical activity, dietary intake, and “mind-body” logs), curriculum materials and up-to-date dietary and physical activity information, and links to other websites of interest private & group mail  Bulletins, Chat rooms, Support group | Website  Individual  Group |  |
| Dale 2009 | Dietary and exercise advice (modest and intensive level) | Usual diet and exercise routine | Experienced dietitian and physical activity instructor  Community, New Zealand | 16 weeks (with a 24 months follow-up)  Individually designed advice. Seen weekly by researchers for weight measurements and a short dietary and exercise assessment. 8 and 12 month encouraging visits to discuss diet and PA | Face to face  Individual |  |
| De Greef 2011 | Cognitive-behavioral therapy | Usual care from GP | Clinical psychologist with a background in behavior change strategies  General practices, Belgium | 12-weeks  One 90-min group counselling session every three weeks | Face to face  Group |  |
| Eakin 2014 | Telephone counselling | Usual care | Counsellor: least bachelor’s level training in nutrition and dietetics; some also dual-degree in exercise physiology. One-month intensive training in study protocols and motivational interviewing  Primary care, Australia | 72 weeks  27 telephone calls over the 18 months (4 initial weekly calls; fortnightly calls for 5 months; monthly calls for 12 months) | Face to face  Individual  Telephone Workbook | Social Cognitive Theory |
| Eriksson 2009 | Exercise sessions and diet counselling | Usual care + information about healthy behaviour | Physiotherapists, dietitian, physician  Primary care, Sweden | 12 weeks  Exercise training and diet counselling 3 sessions/ week x 5, 6 follow-up meeting/ 1. year, 4/ 2. year, 2/3.year. Diet counselling once/year for 3 years | Face to face  Group | Transtheoretical Model of Behavioural Change |
| Fortier 2011 | Brief + intensive physical activity counselling | Brief physical activity counselling (2-4 min) | Physical activity counsellor, BSc in Human Kinetics, certified Fitness consultant  Primary care, Canada | 12 weeks  6 MI sessions over 3 months every 2 weeks, 3 in person and 3 by telephone | Face to face  Individual  Telephone | Self-determination Theory  Social Cognitive Theory |
| Gallagher 2012 | Weight reduction intervention combining exercise, diet and behaviour change strategies | Waiting list, provided with brochures and information that promote exercise for weight loss | Dietician, clinical psychologist, exercise physiologist, cardiac rehabilitation clinical nurse Consultant Hospital, Australia | 16 weeks  Exercise: minimum of two x 60 min exercise sessions per week for 16 weeks. Support group: four x 90-min week 1, 2, 4 and 8 (multidisciplinary team) | Face to face  Group |  |
| Gray 2013 | A weight management programme | Waiting list standard weight loss advice (booklet) | Male community coaches / fieldworkers/health trainers trained to standard protocols  Two Premier League football clubs, Scotland | 12 weeks  Twelve 90 minute, weekly group sessions consisting of classroom-based education + physical activity | Face to face  Group |  |
| Greene 2013 | A health-oriented online social network | Printed lifestyle guidelines | Unclear  Community, USA | 24 weeks  Private mail. Public postings. View other`s postings and compete in PA | Website  Individual  Group |  |
| Griffin | Intensive treatment plus a behaviour change intervention | Intensive treatment  (3x 10 min with GP + and 3 x 15 min with nurse) | Female trained lifestyle facilitators  34 general practices, England | 48 weeks  1 h introductory meeting followed by six 30 min meetings and four brief phone calls | Face to face  Individual  Telephone | Theory of Planned Behavior  Operant learning theory  Control theory |
| Hardcastle 2008 | Standard exercise and nutrition information + individual consultations | Standard exercise and nutrition information | Physical activity specialist and registered dietitian trained in MI  Local Health Centre, UK | 24 weeks  5 x 30 min face-to-face MI counselling sessions over 6-month | Face to face  Individual | Self-determination Theory  Social Cognitive Theory Transtheoretical model of Behavioural Change |
| Ingelstr$ö$m 2014 | CPAP regimen+ behavioral medicine intervention | CPAP regiment+ brief advice to lose weight | Dietitian and physiotherapist with university courses in behavioral medicine  University hospital, Sweden | 24 weeks  8-10 sessions/6 months, gradually reduced in frequency, and 4 sessions/ 9 months | Face to face  Individual | Social Cognitive Theory  Self-determination Theory  Theory of Self-regulation |
| Hemmingsson 2008 | Added social support on walking | Standard care | Hospital staff  Outpatient obesity clinic, University Hospital, Sweden | 18 weeks  Intervention: standard care 5x 2-h/month meetings + 10 added walking promotion meetings | Face to face  Group | Transtheoretical Model of Behavioural Change |
| Hinderliter 2014 | The Dietary Approaches to Stop Hypertension (DASH diet) plus  behavioral weight management | Usual care | Nutritionist  University Medical center, USA | 16 weeks  30-45 min counselling/week.  3 supervised exercise sessions/week | Face to face  Group |  |
| Jakicic 2009 | Intensive lifestyle weight loss intervention | Diabetes support and  education (standard care plus 3education sessions/year) | Unclear  Unclear, USA | 48 weeks  1–6 month: 3 weekly group sessions and one individual meeting/ month  7–12 months: 2 group meetings + one individual session/month | Face to face  Individual  Group |  |
| Janus 2012 | A primary-care based diabetes prevention programme (Life!) | Usual care by GP | Trained health professionals, a physiotherapist or exercise physiologist and a dietitian co-facilitated  Primary health care, Australia | 48 weeks  6 group sessions: 1-5 each 2.weeks and 1 session 8 months after the first | Face to face  Group |  |
| Kuller 2012 | A lifestyle change and weight loss intervention | Health education  (6 seminars the first year + more through 36 months | Nutritionists, exercise physiologists, psychologists  Community, Pennsylvania, USA | 120 weeks  40 visits during 1 first year and a minimum of 12/year 2-3. | Face to face  Group |  |
| Leblanc 2012 | A Health-At-Every-Size (HAES) intervention | A waiting list control (usual lifestyle habits) | Dietitian, clinical psychologist  Community, Canada | 16 weeks  14 weekly sessions were scheduled (13 x 3-h evening sessions and 1 intensive-day session of 6 h) | Face to face  Group |  |
| Liebreich 2009 | The Canadian Diabetes Association's Clinical Practice Guidelines for Physical Activity and Canada's Guide to Physical Activity + The Diabetes NetPLAY intervention | The Canadian Diabetes Association's Clinical Practice Guidelines for Physical Activity and Canada's Guide to Physical Activity | Counsellor with a Bachelor of Science in Physical Education + Masters of Science in Health Promotion  Community, Canada | 12 weeks  Personalized weekly emails, an on-line logbook and message board (forum) | Website  Individual | Social Cognitive Theory |
| Lier 2012 | A preoperative counselling program | Treatment as usual: 2x 4 hours educational  seminars (preoperative and postoperative) | Psychiatrist, psychologist, physiotherapist  Hospital, Norway | 12 weeks  One preoperative session/week for six weeks, three postoperative sessions 6, 12, 24 months after surgery | Face to face  Group |  |
| Logan 2010 | Advice to implement a Mediterranean-style diet using behavioural counselling | Conventional dietetic advice for CHD | Research dietitian  Hospital, UK | 24 weeks  A home visit at week 1 (optional), home visits at months 1, 2 and 4 | Face- to face  Individual  Telephone | Social Cognitive Theory Transtheoretical Model of Behavioural Change |
| Lynch 2014 | Health coaching (acceptance and commitment therapy) | Usual-care (educational brochures) | Nurses, psychologists or health promotion practitioners trained in acceptance and commitment therapy  Community, Australia | 24 weeks  11 sessions/6 months | Face to face  Individual  Telephone | Acceptance and Commitment theory |
| Marcus 2013 | Culturally adapted, Spanish-language, individually tailored,  computer expert system–driven physical activity print-based intervention | A wellness contact control (pamphlets on healthy behaviour) | Researcher  Community, USA | 48 weeks  Print materials by email | Website Individual | Social Cognitive Theory Transtheoretical Model of Behavioural Change |
| Mascola 2009 | Usual medical care plus a brief intervention highlighting the benefits of activity independent of weight-management | Usual care (encouraged to select their own personal health goals and use resources available  in their health-care plan and community) | Pre-professional student volunteers with limited clinical experience trained in MI  Medical centre, USA | 12 weeks  3 in-person consultations followed by up to 4 brief telephone contacts | Face to face  Individual  Telephone | Intertemporal Bargaining Theory |
| Miller 2009 | A group-based 3-month mindful eating intervention | Diabetes self-  management education | Dietitian and social worker with extensive training in mindful meditation  Community, USA | 12 weeks  8 weekly and 2 biweekly 2½ hour group sessions | Face to face  Group | Social Cognitive Theory |
| Morgan 2011 | A weight program | Wait-list control | Male researcher  Community, Australia | 14 weeks  One information session and an free publicly accessible, weight loss website | Face to face  Individual  Website | Social Cognitive Theory |
| Nakade 2012 | Individual-based counselling | Wait-list control | Registered dietitians and exercise instructors  Health doc center, Japan | 48 weeks  Individual counselling (30 minutes) and group sessions about effective exercise (20 minutes) at baseline and at 1, 3, 6 and 9 months | Face to face  Individual  Group | Transtheoretical Model of Behavioural Change |
| Nicklas 2014 | Weight loss intervention with  a self-regulatory intervention to promote physical activity and decrease sedentary behavior | Weight loss intervention without a self-regulatory intervention | RD and an exercise physiologist  Community, USA | 20 weeks  A controlled diet and 4 days/week of aerobic exercise. In addition, the first 6-weeks: 10-15 min individual session/week. Then biweekly sessions during the 5 months’ intervention. During the 5-month follow-up period: brief sessions at 5-week intervals | Face to face  Individual |  |
| Nilsen 2011 | A low-intensity individual lifestyle intervention by a physician combined with an interdisciplinary, group-based approach | Usual care | Dietician, physiotherapist,  ergonomist, nurse, physician using Motivational interviewing techniques  University hospital, Norway | 16 weeks  One day (five hours) /week for six weeks and a new gathering after twelve weeks. An individual 30-minutes consultation completed the intervention one month after the last group meeting. | Face to face  Group  Individual |  |
| Pakiz 2011 | A weight loss intervention | A waiting list | Trained investigators and research staff  Community, USA | 48 weeks  Group sessions weekly for 4 months, and follow-up monthly sessions through 12 months. All intervention subjects also received individualized telephone-based counselling, starting with weekly calls and decreasing infrequency after the first month (every other week for the next 2 months, and once a month thereafter) | Face to face  Group  Individual  Telephone |  |
| Patrick 2011 | 1-year internet-based weight loss intervention for men | A waiting list | Dietitian, physical activity expert, clinical psychologist  Community, USA | 48 weeks  A web-based assessment of diet and physical activity behaviors and weekly tailored Web modules addressing weight-related behaviors | Website  Individual | Social Cognitive Theory |
| Pekmezi 2009 | A Culturally Adapted Physical Activity Intervention for Latinas | A wellness contact control (health information on topics other than physical activity) | Bilingual/bicultural staff  Community, USA | 24 weeks  Monthly mailings of physical activity manuals that were matched to the participants’ current level of motivational readiness and individually tailored computer expert-system feedback reports | Website  Individual | Social Cognitive Theory Transtheoretical Model of Behavioural Change |
| Pettman 2009 | A diet and lifestyle modification program for individuals with  metabolic syndrome | Written copies of the Australian national guidelines for healthy eating and the Australian National PA guidelines | Study coordinator (with health/ nutrition background) and a peer leader/study coordinator (experienced in adult training and  self-management programs)  Community, Australia | 16 weeks  Information and PA sessions for up to 2 h/week | Face to face  Group |  |
| Provencher 2009 | A Health-At-Every-Size (HAES) intervention | A wait-list | Registered dietitian and a clinical  psychologist  Community, Canada | 20 weeks  14 weekly sessions (13 3-hour evening sessions and one intensive 1-day session lasting 6 hours) | Face to face  Group |  |
| Tapper 2009 | Mindfulness-based weight loss intervention for women | Continue with their normal diets | Researcher trained in Mindfulness  Community, England | 15 weeks  Three workshops over three consecutive weeks with a fourth follow-up session approximately 3 months later | Face to face  Group |  |
| Webber 2010 | A motivation-enhanced behavioral weight loss intervention | Standard Intervention | Dietitian trained in MI by the Motivational Interviewing  Network of Trainers  Community, USA | 16 weeks  An initial + a four-week two-hour group weight loss session. The website contained weight loss tips, lesson postings, recipes, a message board feature, links to self-help diet, exercise, behavioral modification resources, on-line self-monitoring report | Face to face  Group  Web based  Individual | Self-Determination Theory |
| Weinstock 2011 | Informatics for Diabetes Education and telemedicine intervention and pedometer use on physical activity (PA) and impairment | Usual care | Bilingual educators and diabetes educator  Primary care, USA | 240 weeks  Home visits (videoconference) every 4–6 weeks for 5 years (Telemedicine) | Web based  Face to face  Individual |  |
| Duda 2014 | An autonomy supportive exercise referral program on physical activity, quality of life  and well-being indicators | A standard exercise referral program | SDT trained Health and fitness advisor  Community, UK | 12 weeks  One-to-one contact, in person or via telephone, with participants on four occasions | Face to face  Individual Telephone | Self-Determination Theory  (Basic Needs Theory) |
| Folta 2009 | A community-based program that improve heart health in midlife and older women | A delayed-intervention control group | Cooperative State Research, Education, and Extension educators at the US Department of Agriculture  Community, USA | 12 weeks  1 hour 2 days per week for 12 weeks | Face to face  Group | Social Cognitive Theory |

*Abbreviations:* PA= physical activity.
