# Supplementary material for: Effective behaviour change techniques for physical activity and healthy eating in overweight and obese adults; systematic review and meta-regression analyses
Source: Int J Behav Nutr Phys Act. 2017 Mar 28;14:42. doi: 10.1186/s12966-017-0494-y (PMC5370453; doi:10.1186/s12966-017-0494-y)
Supplement: Supplementary file 4 — Risk of bias in 48 included studies by first author (DOCX 32 kb) [file 12966_2017_494_MOESM4_ESM.docx]

| **Type of bias** | Adams 2013 | Anderson 2014 | Annesi 2013 | Assunco 2010 | Befort 2008 | Blomfield 2014 | Rejeski 2011 | Carr 2008 | Carr 2013 | Cussler 2008 | Dale 2009 | De Greef 2011 | Eakin 2014 | Eriksson 2009 | Fortier 2011 | Gallanger 2012 | Gray 2013 | Green 2013 | Griffin 2014 | Hardcastle 2008 | Ingelstrom 2014 | Hemmingsson 2008 | Hinderliter 2014 | Jakicic 2009 | Janus 2012 | Kuller 2012 | Leblanc 2012 | Liebreich 2009 | Lier 2012 |
| --- | --- | --- | --- | --- | --- | --- | --- | --- | --- | --- | --- | --- | --- | --- | --- | --- | --- | --- | --- | --- | --- | --- | --- | --- | --- | --- | --- | --- | --- |
| Random sequence generation | L^[[1]](#footnote-1)^ | L | ? | L | L | ? | L | ? | L | ? | ? | L | L | L | L | L | L | ? | L | L | L | ? | L | L | L | L | L | L | L |
| Allocation concealment | ? | L | ? | L | L | ? | L | ? | L | ? | ? | L | L | L | L | L | ? | ? | L | L | ? | ? | L | L | L | L | L | L | L |
| Performance | H | H | H | H | H | H | H | H | H | H | H | H | H | H | H | H | H | H | H | H | H | H | H | H | H | H | H | H | H |
| Blinding of outcome assessment | L | H | H | H | H | H | L | L | L | H | H | H | H  L^[[2]](#footnote-2)^ | H | L | H | H | L | L | H | H  L | L | H | H | H | H | H | H | H |
| Attrition | L | L | ? | L | H | ? | L | H | H | L | L/H^[[3]](#footnote-3)^ | L | L | L | L | L | L | L | L | H | L | L | L | L | H | L | L | L | L |
| Reporting | L | L | L | L | L | L | L | L | L | L | H | L | L | L | L | L | L | L | L | L | L | L | L | H | L | L | L | L | L |

|  | Logan 2009 | Lynch 2014 | Marcus 2013 | Mascola 2009 | Miller 2009 | Morgan 2011 | Nakade 2012 | Nicklas 2014 | Nilsen 2011 | Pakiz 2011 | Patrick 2011 | Pekmezi 2009 | Pettman 2009 | Provencher 2000 | Tapper 2009 | Webber 2010 | Weinstock 2011 | Duda 2014 | Folta 2009 |
| --- | --- | --- | --- | --- | --- | --- | --- | --- | --- | --- | --- | --- | --- | --- | --- | --- | --- | --- | --- |
| Random sequence generation | L | L | L | L | L | L | ? | ? | L | ? | L | L | L | ? | L | ? | ? | L | ? |
| Allocation concealment | L | L | ? | L | ^L^ | L | ? | ? | L | ^?^ | ? | ? | ? | ? | ? | ? | ? | L | L |
| Performance bias | H | H | H | H | H | H | H | H | H | H | H | H | H | H | H | H | H | L | H |
| Blinding of outcome assessment | H | H | H | H | H | H | H  L | L | H | H | H | H | H | H | H | H | H | L | H  L |
| Attrition bias | L/H | L | L | L | L | L | L | L | L | H | L | L | L | H | L | L | ? | L | H |
| Reporting bias | L | L | L | L | L | L | L | L | L | L | L | L | L | L | L | L | L | L | H |

1. L = Low risk;? = Unclear risk; H = high risk [↑](#footnote-ref-1)
2. H score for diet outcome measure and L score for physical activity outcome measure [↑](#footnote-ref-2)
3. L/H means score for short term/long term outcome results [↑](#footnote-ref-3)
