# Supplementary material for: Effective behaviour change techniques for physical activity and healthy eating in overweight and obese adults; systematic review and meta-regression analyses
Source: Int J Behav Nutr Phys Act. 2017 Mar 28;14:42. doi: 10.1186/s12966-017-0494-y (PMC5370453; doi:10.1186/s12966-017-0494-y)
Supplement: Supplementary file 5 — BCTs unique to the intervention and not in the control group coded by The behaviour change technique taxonomy. (DOCX 29 kb) [file 12966_2017_494_MOESM5_ESM.docx]

| BCT | 1.1 | 1.2 | 1.3 | 1.4 | 1.5 | 1.6 | 1.7 | 1.9 | 2.2 | 2.3 | 2.4 | 2.7 | 3.1 | 3.2 | 4.1 | 4.2 | 4.3 | 5.1 | 6.1 | 6.2 | 7.1 | 8.1 | 8.2 | 8.7 | 9.2 | 11.2 | 12.5 | 13.2 | 13.4 |
| --- | --- | --- | --- | --- | --- | --- | --- | --- | --- | --- | --- | --- | --- | --- | --- | --- | --- | --- | --- | --- | --- | --- | --- | --- | --- | --- | --- | --- | --- |
| Adams 2013 |  |  |  |  |  |  |  |  | x |  |  |  |  |  |  |  |  |  |  |  |  | x |  | x |  |  |  |  |  |
| Anderson 2014 | x | x | x | x | x |  | x |  | x | x | x | x | x |  |  |  |  |  |  |  |  |  |  | x | x |  | x |  | x |
| Annesi 2013 |  |  |  |  |  |  |  |  |  | x | x |  |  |  | x | x | x |  |  |  |  |  |  |  |  | x |  |  |  |
| Assuncao 2010 | x |  | x | x |  |  |  |  |  |  |  |  | x |  |  |  |  |  | x |  |  |  | x |  |  |  |  |  |  |
| Befort 2008 |  |  |  |  |  |  |  | x |  |  |  |  |  |  |  |  |  |  |  |  |  |  |  |  | x |  |  |  | x |
| Blomfield 2014 | x |  | x |  | x | x | x |  | x | x | x | x |  |  | x |  |  | x |  |  |  |  |  |  |  |  | x |  |  |
| Rejeski 2011 | x | x | x |  |  |  |  |  |  | x | x | x | x |  |  | x |  |  | x |  |  |  |  |  |  |  | x |  |  |
| Carr 2008 | x |  |  |  | x |  |  |  | x |  |  |  | x |  | x |  |  | x | x |  | x | x | x |  |  | x |  | x |  |
| Carr 2013 | x |  |  |  |  |  |  |  | x | x |  |  | x |  | x |  |  | x |  | x | x |  |  |  |  |  | x | x |  |
| Cussler 2008 |  |  |  |  |  |  |  |  |  | x | x |  |  |  |  |  |  |  |  |  | x |  |  |  |  |  |  |  |  |
| Dale 2009 | x |  | x | x |  |  |  |  |  | x |  | x |  | x | x |  |  | x | x |  |  | x |  |  |  |  | x |  |  |
| De Greef 2011 | x | x |  | x |  |  |  |  |  |  |  |  | x |  | x |  | x | x |  |  |  |  |  |  | x |  |  | x |  |
| Eakin 2014 | x | x | x | x | x | x | x |  | x | x | x | x | x |  |  |  |  |  | x |  | x |  |  | x |  |  | x |  |  |
| Eriksson 2009 | x | x | x | x |  |  |  |  |  |  |  | x | x | x |  |  |  |  | x |  |  | x |  | x | x |  |  |  |  |
| Fortier 2011 |  | x | x | x | x |  |  |  |  | x |  |  | x |  |  |  |  |  |  |  |  |  |  | x | x | x | x |  | x |
| Gallagher 2012 | x | x |  | x |  |  |  |  |  | x |  |  | x |  | x |  |  | x | x |  |  | x | x |  |  |  | x |  |  |
| Gray 2013 | x |  | x | x |  |  |  | x | x | x | x | x | x |  | x | x | x |  |  |  |  | x | x | x |  | x | x |  |  |
| Greene 2013 |  |  |  |  |  |  |  |  |  | x | x |  |  |  |  |  |  |  |  | x | x |  |  |  |  |  | x |  |  |
| Griffin 2014 |  | x |  |  |  |  |  |  |  |  |  |  |  |  |  |  |  |  |  |  | x |  |  |  |  |  | x |  |  |
| Hardcastle 2008 |  |  |  | x | x |  | x | x |  |  |  |  | x |  |  |  |  |  |  |  |  |  |  |  | x |  |  |  |  |
| Ingelstr$\boldsymbol{ö}$m 2014 | x | x | x | x | x |  |  | x | x | x |  |  |  |  | x | x |  |  |  |  |  |  |  | x |  |  |  |  | x |
| Hemmingsson 2008 |  |  |  |  |  | x |  |  |  |  |  |  |  | x |  |  |  |  | x |  |  |  |  |  |  |  |  |  |  |
| Hinderliter 2014 | x |  |  | x |  |  |  |  |  | x |  |  | x |  |  |  |  |  | x |  |  | x |  |  |  |  |  | x |  |
| Jakicic 2009 | x | x | x | x |  |  |  |  |  | x |  | x | x | x |  |  |  |  |  | x |  |  | x | x |  | x | x |  |  |
| Janus 2012 | x |  | x | x |  |  |  |  |  |  |  |  | x |  | x |  |  | x |  |  |  |  |  |  |  |  |  |  |  |
| Kuller 2012 | x | x |  | x |  |  |  |  |  | x |  |  | x |  |  |  |  |  |  |  |  |  |  | x |  |  |  | x |  |
| Leblanc 2012 |  |  | x |  |  |  |  |  |  |  |  |  | x |  | x | x |  | x |  |  |  |  |  |  |  | x |  |  | x |
| Liebreich 2009 | x |  |  |  |  |  |  |  | x | x |  |  | x |  |  |  |  |  |  |  |  |  |  |  |  |  |  |  |  |
| Lier 2012 | x | x |  | x |  |  |  |  |  | x |  |  | x |  | x |  |  |  |  | x |  | x |  |  |  | x |  |  |  |
| Logan 2010 | x |  |  | x |  |  |  |  |  |  |  |  | x |  |  |  |  |  |  |  |  |  |  | x |  |  |  |  |  |
| Lynch 2014 | x | x |  | x | x |  |  |  |  | x |  |  | x |  |  |  |  |  |  |  | x |  |  |  |  | x | x |  | x |
| Marcus 2013 | x | x |  |  |  |  |  |  | x | x |  |  | x |  |  |  |  |  |  | x | x |  |  |  |  |  | x |  |  |
| Mascola 2009 |  |  |  |  |  |  |  |  |  | x |  |  |  |  |  |  |  |  |  |  |  |  |  | x |  | x |  |  | x |
| Miller 2009 |  |  |  |  |  |  |  |  |  |  |  |  |  |  |  | x | x |  |  |  |  | x | x |  |  | x |  |  |  |
| Morgan 2011 | x |  |  |  |  |  |  |  | x | x | x | x | x |  | x |  |  | x |  |  |  |  |  | x |  |  |  |  |  |
| Nakade 2012 | x |  |  |  | X |  |  |  | x | x | x |  | x |  | x |  |  | x | x |  |  | x |  | x |  |  |  |  |  |
| Nicklas 2014 |  | x |  |  | X | x |  |  |  |  |  |  | x |  |  |  |  |  |  |  |  |  | x | x |  |  |  |  |  |
| Nilsen 2011 |  |  |  |  |  |  |  |  |  |  |  |  | x |  |  |  |  |  | x |  |  | x |  |  |  |  |  |  |  |
| Pakiz 2011 | x | x | x |  | X |  |  | x |  | x |  |  | x |  | x |  |  | x |  |  |  | x |  |  | x | x |  |  |  |
| Patrick 2011 | x |  |  | x | X | x |  |  | x | x |  |  | x |  | x |  |  | x |  |  |  |  | x | x |  |  | x |  |  |
| Pekmezi 2009 | x | x |  |  |  |  |  |  | x | x |  |  | x |  | x |  |  | x |  | x |  |  |  |  |  |  | x |  |  |
| Pettman 2009 | x | x |  | x |  |  |  |  | x | x |  |  |  | x |  | x |  |  | x |  |  | x |  |  |  | x | x |  |  |
| Provencher 2009 |  |  | x |  |  |  |  |  |  |  |  |  |  |  | x |  | x | x | x |  |  | x |  |  |  | x |  | x | x |
| Tapper 2009 | x |  | x |  |  |  |  |  |  |  |  |  | x |  |  |  | x |  |  |  |  |  |  |  |  | x | x | x | x |
| Webber 2010 |  |  |  |  | X |  |  |  |  |  |  |  |  |  |  |  |  |  |  |  |  |  |  |  | x |  |  |  |  |
| Weinstock 2011 |  |  |  |  |  |  | x |  | x |  |  | x |  |  | x |  |  | x |  |  |  |  |  |  |  |  |  |  |  |
| Duda 2014 |  | x |  | x | X |  | x |  |  |  |  |  |  | x |  |  |  |  |  |  |  |  |  |  | x |  |  | x |  |
| Folta 2009 | x |  |  |  |  |  |  |  |  | x |  |  |  |  | x |  |  | x | x |  |  | x |  |  |  |  |  |  |  |
| Times | 30 | 19 | 16 | 21 | 14 | 5 | 6 | 5 | 16 | 28 | 10 | 10 | 30 | 6 | 20 | 7 | 6 | 16 | 14 | 6 | 8 | 15 | 8 | 15 | 9 | 14 | 18 | 8 |  |
| BCT | **1.1** | **1.2** | **1.3** | **1.4** | **1.5** | **1.6** | **1.7** | **1.9** | **2.2** | **2.3** | **2.4** | **2.7** | **3.1** | **3.2** | **4.1** | **4.2** | **4.3** | **5.1** | **6.1** | **6.2** | **7.1** | **8.1** | **8.2** | **8.7** | **9.2** | **11.2** | **12.5** | **13.2** | **13.4** |

*Abbreviations and symbols:* BCT: Behaviour change technique identified. Here presented by the difference in BCTs between intervention and control group. Behaviour change techniques are identified using the Behaviour Change Techniques Taxonomy version 1. Results of a systematic review of 48 studies with ≥ 12 weeks’ duration for adults (mean age ≥ 40 years and with a mean BMI ≥ 30) published from January 2007 to October 2014.
