# Supplementary material for: Effective behaviour change techniques for physical activity and healthy eating in overweight and obese adults; systematic review and meta-regression analyses
Source: Int J Behav Nutr Phys Act. 2017 Mar 28;14:42. doi: 10.1186/s12966-017-0494-y (PMC5370453; doi:10.1186/s12966-017-0494-y)
Supplement: Supplementary file 6 — Results from simple linear meta-regression analysis of short term reports of PA and diet interventions. (DOCX 35 kb) [file 12966_2017_494_MOESM6_ESM.docx]

**Additional file 6: Table 4 Results from simple linear meta-regression analysis of short term reports of PA and diet interventions¹**

| **50 trials at short term** | | | | | | | | | |
| --- | --- | --- | --- | --- | --- | --- | --- | --- | --- |
| **Covariate** | **Classification** | **Trials N** | **Adj. R² %** | **Effect size** | **95% CI** | **I² (%)** | **b** | **95% CI** | **P value** |
| None | Overall effect PA + Diet short term | 50 |  | 0.367 | (0.257, 0.477) | 71.3 |  |  | **< 0.001** |
| Type of behaviour | Physical activity | 30 |  | 0.358 | (0.241, 0.475) | 72.2 |  |  | **< 0.001** |
|  | Diet | 20 |  | 0.409 | (0.196, 0.623) | 86.7 |  |  | **< 0.001** |
| Risk of bias (High risk or unclear risk)² | |  |  |  |  |  |  |  |  |
|  | Random sequence |  | -3.74 |  |  | 71.34 | -0.034 | (-0.330, 0.264) | 0.818 |
|  | Allocation concealment |  | -4.00 |  |  | 71.90 | 0.075 | (-0.190, 0.341) | 0.571 |
|  | Performance bias (Blinding participants + personnel) |  | 0.51 |  |  | 70.39 | 0.400 | (-0.389,1.189) | 0.313 |
|  | Detection bias (blinding assessment) |  | -2.07 |  |  | 71.65 | -0.099 | (-0.414, 0.215) | 0.528 |
|  | Attrition bias |  | -2.58 |  |  | 70.76 | -0.068 | (-0.367, 0.230) | 0.648 |
|  | Reporting bias |  | 18.51 |  |  | 68.65 | 0.670 | (0.100, 1.240) | **0.022** |
| Study characteristics s at short term³ | |  |  |  |  |  |  |  |  |
| Theory based | yes |  | -4.07 |  |  | 71.64 | 0.014 | (0.020, 0.135) | 0.881 |
| Method based | 0=MI + SDT |  |  |  |  |  | 0.000 | reference |  |
|  | 1=ACT+ CT+ HAES + Mindful or other |  | -5.53 |  |  | 72.14 | 0.183 | (-0.148, 0.513) | 0.272 |
|  | 2= Unclear |  |  |  |  |  | 0.179 | (0.164, 0.522) | 0.299 |
| Single or multiple domain intervention |  |  | -3.69 |  |  | 71.37 | 0.037 | (0.233, 0.307) | 0.785 |
| Number of BCTs unique in intervention group |  |  | 23.83 |  |  | 65.29 | 0.033 | (0.008, 0.059) | **0.012** |
| Total number BCTs intervention + control group |  |  | 8.85 |  |  | 68.94 | 0.017 | (-0.006, 0.040) | 0.136 |
| Type of outcome data | Objective or self-report measure |  | 2.49 |  |  | 70.03 | -0.162 | (-0.467, 0.142) | 0.290 |
| Intervention duration | Weeks |  | -5.19 |  |  | 71.21 | -0.000 | (-0.003, 0.003) | 0.916 |
| Source of delivery | 0= Not health professionals or unclear |  |  |  |  |  | 0.000 | reference |  |
|  | 1= Profess. trained in behaviour change |  | 6.48 |  |  | 69.46 | -0.283 | (-0.607, 0.040) | ***0.085*** |
|  | 2= Health profess. other than 1 |  |  |  |  |  | -0.201 | (-0.550, 0.148) | 0.252 |
| Format of delivery | 0=Individually based only: Face to face or Web |  |  |  |  |  | 0.000 | reference |  |
|  | 1=Group based or mixed (Individual +group+ web) |  | -5.27 |  |  | 71.70 | 0.087 | (-0.184, 0.357) | 0.522 |
| Treatment setting | 0= Community or Workplace |  |  |  |  |  | 0.000 | reference |  |
|  | 1= Primary care or Hospital |  | -4.08 |  |  | 71.87 | 0.053 | (-0.217, 0.322 | 0.696 |

| **BCTs at short term⁴** | **Different BCT** | **Measure of agreement**  **Kappa** | **% agreement** | **Adj. R² %** | **I² (%)** | **b** | **95% CI** | **P value** |
| --- | --- | --- | --- | --- | --- | --- | --- | --- |
| 1.1 Goal setting (behaviour) | 30 | 0.602 | 81.4 | 49.17 | 59.29 | 0.480 | (0.257, 0.705) | **< 0.001** |
| 1.2 Problem solving | 19 | 0.532 | 76.7 | -0.71 | 70.41 | 0.127 | (-0.136, 0.389) | 0.338 |
| 1.3 Goal setting (outcome) | 16 | 0.705 | 88.3 | 2.52 | 69.88 | 0.180 | (-0.089, 0.449) | 0.184 |
| 1.4 Action planning | 21 | 0.534 | 76.8 | -3.50 | 71.72 | 0.107 | (-0.155, 0.369) | 0.417 |
| 1.5 Review behaviour goals | 14 | 0.239 | 72.1 | -0.65 | 75.72 | 0.217 | (-0.160, 0.651) | 0.313 |
| 1.6 Discrepancy between current behaviour and goal | 5 | 0.541 | 93.1 | -2.53 | 71.14 | 0.071 | (-0.299, 0.441) | 0.700 |
| 1.7 Review outcome goal(s) | 6 | 0.449 | 90.7 | -3.24 | 70.92 | -0.092 | (-0.410, 0.224) | 0.559 |
| 1.9 Commitment | 5 | 0.452 | 90.7 | -3.15 | 71.75 | 0.141 | (-0.244, 0.527) | 0.466 |
| 2.2 Feedback on behaviour | 16 | 0.428 | 72.1 | 4.44 | 71.16 | 0.219 | (-0.040, 0.479) | ***0.096*** |
| 2.3 Self-monitoring of behaviour | 28 | 0.718 | 86..0 | 35.30 | 62.49 | 0.398 | (0.164, 0.632) | **0.001** |
| 2.4 Self-monitoring of outcome(s) of behaviour | 10 | 0.439 | 83.7 | 6.83 | 68.81 | 0.192 | (-0.116, 0.500) | 0.215 |
| 2.7 Feedback on outcome of behaviour | 10 | 0.487 | 83.7 | 11.99 | 70.67 | 0.243 | (-0.040, 0.527) | ***0.091*** |
| 3.1 Social support (unspecified) | 30 | 0.223 | 62.8 | 0.93 | 69.40 | 0.097 | (-0.178, 0.371) | 0.483 |
| 3.2 Social support (practical) | 6 | 0.534 | 93.1 | -4.22 | 71.62 | -0.019 | (-0.443, 0.407) | 0.943 |
| 4.1 Instruction how to perform the behaviour | 20 | 0.348 | 67.5 | -2.70 | 71.91 | 0.168 | (-0.095, 0.432) | 0.205 |
| 4.2 Information on antecedent | 7 | 0.292 | 83.8 | -4.02 | 71.87 | -0.005 | (-0.365, 0.355) | 0.978 |
| 4.3 Re-attribution | 6 | 0.325 | 86.1 | -2.75 | 71.81 | -0.131 | (-0.531, 0.269) | 0.514 |
| 5.1 Information about health consequences | 16 | 0.579 | 79.1 | -3.64 | 71.83 | 0.064 | (-0.215, 0.342) | 0.648 |
| 6.1 Demonstration of the behaviour | 14 | 0.546 | 79.1 | 11.93 | 68.64 | 0.244 | (-0.035, 0.523) | ***0.085*** |
| 6.2 Social comparison | 6 | 0.422 | 86.1 | -1.41 | 71.26 | 0.191 | (-0.351, 0.733) | 0.548 |
| 7.1 Prompts/cues | 8 | 0.366 | 83.7 | -0.31 | 70.71 | 0.153 | (-0.186, 0.492) | 0.368 |
| 8.1 Behavioural practice /Rehearsal | 15 | 0.514 | 79.0 | 3.80 | 70.36 | 0.193 | (-0.109, 0.495) | 0.205 |
| 8.2 Behaviour substitution | 8 | 0.271 | 83.8 | -2.35 | 71.79 | -0.130 | (-0.480, 0.220) | 0.485 |
| 8.7 Graded tasks | 15 | 0.475 | 79.1 | 2.95 | 69.79 | 0.210 | (-0.062, 0.482) | 0.127 |
| 9.2 Pros and cons | 9 | 0.758 | 93.1 | 4.77 | 70.82 | -0.252 | (-0.542, 0.038) | ***0.087*** |
| 11.2 Reduce negative emotions | 14 | 0.606 | 88.3 | 7.08 | 71.01 | -0.237 | (-0.523, 0.492) | 0.102 |
| 12.5 Adding objects to the environment | 18 | 0.473 | 74.4 | 10.67 | 67.33 | 0.194 | (-0.061, 0.450) | 0.133 |
| 13.2 Framing/ reframing | 8 | 0.372 | 83.7 | -2.97 | 71.90 | 0.115 | (-0.266, 0.496) | 0.546 |
| 13.4 Valued self-identity | 9 | 0.271 | 83.8 | -1.68 | 71.85 | -0.076 | (-0.383, 0.230) | 0.617 |

*Abbreviations and symbols:* BCT: behaviour change technique; PA: physical activity; b: estimated meta-regression coefficient; CI: confidence interval; Adj. R²: adjusted proportion of between study variance explained by predictors. ¹) Pooled estimates of physical activity and diet intervention’s outcome reports from 48 studies. Simple linear meta-regression of pooled estimates of 30 physical activity and 20 diet intervention’s outcome reports. Short term represents post-intervention reports ≤6 months. ²) High and unclear risk of reporting bias versus low risk. ³) MI = Motivational Interviewing; SDT = Self-Determination theory based interventions; ACT = Acceptance and commitment therapy; CT = Cognitive therapy; HAES = Health-at-every-size approach; Mindful= Mindful based intervention program. ⁴) The difference of BCTs between intervention and control group contains this BCT, compared to studies not having this difference.
