# Supplementary material for: Effective behaviour change techniques for physical activity and healthy eating in overweight and obese adults; systematic review and meta-regression analyses
Source: Int J Behav Nutr Phys Act. 2017 Mar 28;14:42. doi: 10.1186/s12966-017-0494-y (PMC5370453; doi:10.1186/s12966-017-0494-y)
Supplement: Supplementary file 7 — Results from simple linear meta-regression analysis of long term reports of PA and diet interventions. (DOCX 33 kb) [file 12966_2017_494_MOESM7_ESM.docx]

| **Additional file 7: Table 5 Results from simple linear meta-regression analysis of long term reports of PA and diet interventions¹** | | | | | | | | | |
| --- | --- | --- | --- | --- | --- | --- | --- | --- | --- |
| **32 trials at long term** |  |  |  |  |  |  |  |  |  |
| **Covariate** | **Classification** | **Trials N** | **Adj. R² %** | **Effect size** | **95% CI** | **I² (%)** | **b** | **95% CI** | **P value** |
| None | Overall effect PA + Diet long term | 32 |  | 0.240 | ( 0.150, 0.330) | 59.4 |  |  | **< 0.001** |
| Type of behaviour | Physical activity | 17 |  | 0.254 | (0.126, 0.383) | 79.6 |  |  | **<0.001** |
|  | Diet | 15 |  | 0.191 | (0.072, 0.310) | 53.8 |  |  | **<0.001** |
| Risk of bias (High risk or unclear risk) | |  |  |  |  |  |  |  |  |
|  | Random sequence |  | 1.85 |  |  | 57.19 | -0.129 | (-0.359, 0.101) | 0.261 |
|  | Allocation concealment |  | 2.23 |  |  | 55.93 | -0.105 | (-0.305, 0.095) | 0.293 |
|  | Performance bias (Blinding part. + person) |  |  |  |  |  |  |  |  |
|  | Detection bias (blinding assessment) |  | -9.42 |  |  | 59.75 | -0.048 | (-0.265, 0.170) | 0.657 |
|  | Attrition bias |  | 6.02 |  |  | 56.53 | -0.136 | (-0.368, 0.097) | 0.242 |
|  | Reporting bias |  | 26.77 |  |  | 39.14 | 0.235 | (-0.054, 0.524) | 0.107 |
| Study characteristics at long term² | |  |  |  |  |  |  |  |  |
| Theory based | Yes |  | 0.47 |  |  | 52.42 | -0.039 | (-0.218, 0.140) | 0.661 |
| Method based | 0=MI + SDT |  |  |  |  |  | 0.000 | reference |  |
|  | 1=ACT+ CT+ HAES + Mindful or other |  | 57.54 |  |  | 31.15 | -0.303 | (-0.500, -0.105) | **0.004** |
|  | 2= Unclear |  |  |  |  |  | -0.199 | (-0.372, -0.026) | **0.026** |
| Single or multiple domain intervention |  |  | 4.01 |  |  | 52.77 | -0.077 | (-0.253, 0.099) | 0.380 |
| Number of BCTs unique to the intervention group |  |  | 54.23 |  |  | 28.79 | 0.028 | (0.012, 0.044) | **0.001** |
| Total number BCT in intervention and control group |  |  | 61.26 |  |  | 23.29 | 0.030 | (0.014, 0.046) | **0.001** |
| Type of outcome data | 1= Objective outcome |  | -9.69 |  |  | 59.56 | -0.046 | (-0.253, 0.161) | 0.652 |
|  | 2= Subjective outcome |  |  |  |  |  | 0.000 | reference |  |
| Intervention duration | Weeks |  | -7.46 |  |  | 60.65 | 0.001 | (-0.001, 0.003) | 0.272 |
| Source of delivery | 0= Not health professionals or unclear |  |  |  |  |  | 0.000 | reference |  |
|  | 1= Profess. trained in behaviour change |  | 5.91 |  |  | 44.99 | -0.128 | (-0.395, 0.138) | 0.333 |
|  | 2= Health profess. other than 1 |  |  |  |  | 44.99 | -0.080 | (-0.349, 0.188) | 0.545 |
| Treatment setting | 0= Community or Workplace |  |  |  |  |  |  |  |  |
|  | 1= Primary care or Hospital |  | -7.07 |  |  | 59.36 | 0.020 | (-0.170, 0.210) | 0.828 |
| Format of delivery | 0=Individually based only: Face to face or Web |  |  |  |  |  | 0.000 | reference |  |
|  | 1=Group based or mixed (Individual +group+ web) |  | 6.00 |  |  | 25 | -0.096 | (-0.322,0.131) | 0.396 |

| **BCTs at long term³** | **Different BCT** | **Adj. R² %** | **I² (%)** | **b** | **95% CI** | **P value** |
| --- | --- | --- | --- | --- | --- | --- |
| 1.1 Goal setting (behaviour) | 30 | 38.51 | 43.56 | 0.228 | (0.056, 0.400) | **0.011** |
| 1.2 Problem solving | 19 | 25.06 | 47.16 | 0.161 | (-0.005, 0.327) | ***0.057*** |
| 1.3 Goal setting (outcome) | 16 | 53.15 | 30.78 | 0. 256 | (0.095, 0.416) | **0.003** |
| 1.4 Action planning | 21 | 15.18 | 50.41 | 0.125 | (-0.049, 0.299) | 0.152 |
| 1.5 Review behaviour goals | 14 | 19.80 | 62.96 | -0.319 | (-0.678, 0.040) | ***0.078*** |
| 1.6 Discrepancy between current behaviour and goal | 5 | -6.53 | 60.70 | 0.114 | (-0.133, 0.360) | 0.354 |
| 1.7 Review outcome goal(s) | 6 | -6.77 | 60.64 | 0.115 | (-0.098, 0.327) | 0.279 |
| 1.9 Commitment | 5 | 9.58 | 57.18 | -0.268 | (-0.639, 0.107) | 0.156 |
| 2.2 Feedback on behaviour | 16 | -9.32 | 60.37 | 0.131 | (-0.056, 0.318) | 0.163 |
| 2.3 Self-monitoring of behaviour | 28 | 30.81 | 47.09 | 0.184 | (0.009, 0.3609) | **0.040** |
| 2.4 Self-monitoring of outcome(s) of behaviour | 10 | -3.50 | 60.72 | 0.117 | (-0.074, 0.307) | 0.220 |
| 2.7 Feedback on outcome of behaviour | 10 | 43.78 | 35.35 | 0.249 | (0.085, 0.412) | **0.004** |
| 3.1 Social support (unspecified) | 30 | 21.59 | 50.86 | 0.192 | (-0.011, 0.394) | ***0.063*** |
| 3.2 Social support (practical) | 6 | 10.09 | 43.75 | 0.137 | (-0.132, 0.405) | 0.307 |
| 4.1 Instruction on how to perform the behaviour | 20 | 2.24 | 55.37 | -0.087 | (-0.274, 0.100) | 0.350 |
| 4.2 Information on antecedent | 7 | -3.06 | 60.47 | 0.215 | (-0.258, 0.689) | 0.361 |
| 4.3 Re-attribution | 6 | -2.87 | 60.28 | -0.129 | (-0.732, 0.475) | 0.666 |
| 5.1 Information about health consequences | 16 | -3.16 | 57.10 | -0.036 | (-0.230, 0.157) | 0.705 |
| 6.1 Demonstration of the behaviour | 14 | -8.72 | 60.23 | 0.070 | (-0.120, 0.259) | 0.459 |
| 6.2 Social comparison | 6 | 7.34 | 43.24 | 0.098 | (-0.205, 0.402) | 0.513 |
| 7.1 Prompts/cues | 8 | -3.79 | 57.57 | -0.031 | (-0.236, 0.173) | 0.755 |
| 8.1 Behavioural practice /rehearsal | 15 | 5.53 | 55.82 | -0.121 | (-0.317, 0.074) | 0.214 |
| 8.2 Behaviour substitution | 8 | 3.33 | 48.03 | 0.096 | (-0.140, 0.332) | 0.412 |
| 8.7 Graded tasks | 15 | 37.10 | 41.31 | 0.203 | (0.043, 0.363) | **0.014** |
| 9.2 Pros and cons | 9 | -5.45 | 59.70 | -0.030 | (-0.276, 0.217) | 0.810 |
| 11.2 Reduce negative emotions | 14 | -2.88 | 49.22 | 0.035 | (-0.195, 0.265) | 0.759 |
| 12.5 Adding objects to the environment | 18 | 12.72 | 49.94 | 0.182 | (0.010, 0.354) | **0.039** |
| 13.2 Framing/ reframing | 8 | -5.58 | 60.71 | 0.050 | (-0.253, 0.353) | 0.738 |
| 13.4 Valued self-identity | 9 | -5.86 | 60.71 | 0.092 | (-0.151, 0.334) | 0.447 |

*Abbreviations and symbols:* BCT: behaviour change technique; PA: physical activity; ß: estimated meta-regression coefficient; CI: confidence interval; Adj. R²: adjusted proportion of between study variance explained by predictors. ¹) Simple linear meta-regression of pooled estimates of 17 physical activity and 15 diet intervention’s outcome reports from 48 studies. Long term outcome reports represented last follow-up, ≥12 months. ²) High and unclear risk of reporting bias versus low risk; MI = Motivational Interviewing; SDT = Self-Determination theory based interventions; ACT = Acceptance and commitment therapy; CT = Cognitive therapy; HAES = Health-at-every-size approach; Mindful= Mindful based intervention program. ³) The difference of BCTs between intervention and control group contains this BCT, compared to studies not having this difference.
