# Supplementary material for: Effective behaviour change techniques for physical activity and healthy eating in overweight and obese adults; systematic review and meta-regression analyses
Source: Int J Behav Nutr Phys Act. 2017 Mar 28;14:42. doi: 10.1186/s12966-017-0494-y (PMC5370453; doi:10.1186/s12966-017-0494-y)
Supplement: Supplementary file 8 — Funnel plot short term. (DOCX 15 kb) [file 12966_2017_494_MOESM8_ESM.docx]

# Additional file 8:

**Figure 4** Funnel plot of 50 outcome reports at short term (≤ 6 months) from diet and physical activity interventions for overweight and obese adults from January 2007 to October 2014
