# Supplementary material for: Effective behaviour change techniques for physical activity and healthy eating in overweight and obese adults; systematic review and meta-regression analyses
Source: Int J Behav Nutr Phys Act. 2017 Mar 28;14:42. doi: 10.1186/s12966-017-0494-y (PMC5370453; doi:10.1186/s12966-017-0494-y)
Supplement: Supplementary file 9 — Funnel plot long term. (DOCX 15 kb) [file 12966_2017_494_MOESM9_ESM.docx]

# Additional file 9:

**Figure 5** Funnel plot of 32 outcome reports at long term (≥ 12 months) from diet and physical activity interventions for overweight and obese adults from January 2007 to October 2014
